# Novel Dicarboximide BK124.1 Breaks Multidrug Resistance and Shows Anticancer Efficacy in Chronic Myeloid Leukemia Preclinical Models and Patients’ CD34^+^/CD38^−^ Leukemia Stem Cells

**DOI:** 10.3390/cancers14153641

**Published:** 2022-07-27

**Authors:** Iga Stukan, Marek Gryzik, Grażyna Hoser, Andrew Want, Wioleta Grabowska-Pyrzewicz, Mikolaj Zdioruk, Mariola Napiórkowska, Marcin Cieślak, Karolina Królewska-Golińska, Barbara Nawrot, Grzegorz Basak, Urszula Wojda

**Affiliations:** 1 Laboratory of Preclinical Testing of Higher Standard, Nencki Institute of Experimental Biology of Polish Academy of Sciences, 3 Pasteur Street, 02-093 Warsaw, Poland; iga.stukan@pum.edu.pl (I.S.); m.gryzik@nencki.edu.pl (M.G.); grazyna.hoser@cmkp.edu.pl (G.H.); a.want@nencki.edu.pl (A.W.); w.grabowska@nencki.edu.pl (W.G.-P.); myko.zdioruk@gmail.com (M.Z.); 2 Centre of Postgraduate Medical Education, 01-813 Warsaw, Poland; 3 Department of Biochemistry, Medical University of Warsaw, 02-097 Warsaw, Poland; mariola.napiorkowska@wum.edu.pl; 4 Centre of Molecular and Macromolecular Studies, Polish Academy of Sciences, 90-363 Lodz, Poland; marcin@cbmm.lodz.pl (M.C.); kkrolews@cbmm.lodz.pl (K.K.-G.); bnawrot@cbmm.lodz.pl (B.N.); 5 Department of Hematology, Transplantation and Internal Medicine, Medical University of Warsaw, 02-097 Warsaw, Poland; grzegorz.basak@wum.edu.pl

**Keywords:** chemotherapy, multidrug resistance, MDR1, P-glycoprotein, leukemia stem cells, chronic myeloid leukemia, apoptosis, cancer, drug resistance, novel therapy

## Abstract

**Simple Summary:**

Chemotherapy is a first line treatment in many cancer types, but the constant exposition to chemotherapeutics often leads to therapy resistance. An example is chronic myeloid leukemia that, due to the use of tyrosine kinase inhibitors such as imatinib, remains manageable, however incurable. Overall, 20–25% of imatinib responders develop secondary resistance, and among them, 20–40% is due to mechanisms such as expression of P-glycoprotein (MDR1) or leukemia stem cells’ mechanisms of survival and cancer regrowth. This study provides the first evidence from animal and cellular models that this resistance can be overcome with the novel dicarboximide BK124.1. The compound causes no visible toxicity in mice, and has proper pharmacokinetics for therapeutic applications. It was efficient against both multidrug resistant CML blasts and CD34^+^/CD38^−^ leukemia stem cells coming from CML patients. Future development of BK124.1 could offer curative treatment of CML and of other cancers resistant or intolerant to current chemotherapy.

**Abstract:**

The search is ongoing for new anticancer therapeutics that would overcome resistance to chemotherapy. This includes chronic myeloid leukemia, particularly suitable for the studies of novel anticancer compounds due to its homogenous and well-known genetic background. Here we show anticancer efficacy of novel dicarboximide denoted BK124.1 (C_31_H_37_ClN_2_O_4_) in a mouse CML xenograft model and in vitro in two types of chemoresistant CML cells: MDR1 blasts and in CD34^+^ patients’ stem cells (N = 8) using immunoblotting and flow cytometry. Intraperitoneal administration of BK124.1 showed anti-CML efficacy in the xenograft mouse model (N = 6) comparable to the commonly used imatinib and hydroxyurea. In K562 blasts, BK124.1 decreased the protein levels of BCR-ABL1 kinase and its downstream effectors, resulting in G2/M cell cycle arrest and apoptosis associated with FOXO3a/p21^waf1/cip1^ upregulation in the nucleus. Additionally, BK124.1 evoked massive apoptosis in multidrug resistant K562-MDR1 cells (IC_50_ = 2.16 μM), in CD34^+^ cells from CML patients (IC_50_ = 1.5 µM), and in the CD34^+^/CD38^−^ subpopulation consisting of rare, drug-resistant cancer initiating stem cells. Given the advantages of BK124.1 as a potential chemotherapeutic and its unique ability to overcome BCR-ABL1 dependent and independent multidrug resistance mechanisms, future development of BK124.1 could offer a cure for CML and other cancers resistant to present drugs.

## 1. Introduction

Chemotherapy remains a first line of treatment used in multiple cancer types. Unfortunately, the rise of resistance to the given drug, often regardless of its specificity, remains one of the main challenges faced in the field of oncology [1]. There is an ongoing search for new therapeutics that would overcome the resistance or intolerance. An example is chronic myeloid leukemia (CML) that in most cases cannot be completely cured without the constant use of tyrosine kinase inhibitors [2,3,4]. This type of leukemia is relatively homogenous genetically and phenotypically compared to other types of cancer [5]. This unique ability creates a convenient model for the first mechanistic studies of novel anticancer compounds. CML is characterized by the uncontrolled proliferation of myeloid precursor cells in the bone marrow and their accumulation in the blood. The disease mainly affects elderly adults and is diagnosed in 1–2 people per 100,000. CML represents 15% of all newly diagnosed adult leukemia cases and 0.5% of all cancer cases in the USA [6].

CML progresses through three distinct clinical phases: an initial chronic phase, accelerated, and finally blast phases, broadly defined by the proportion of blast cells found in blood or bone marrow. In the past CML was treated with nonspecific agents, such as busulfan or hydroxyurea [7]. The breakthrough in CML treatment was the introduction of Tyrosine Kinase Inhibitors (TKIs), such as the first generation imatinib, targeting the main CML oncogene—constitutively activated BCR-ABL1 kinase. The kinase results from t(9;22) chromosome translocation in a hematopoietic stem cell [8] and promotes proliferation through downstream signaling pathways involving STAT, Akt, and mTOR kinases. Imatinib enhanced the 10-year survival rate of CML patients from 20% to 80–90% [9].

The prolonged imatinib intake can cause point mutations in BCR-ABL1, such as T315I mutation in ATP-binding site. This leads to insensitivity to first and second generation TKIs [10,11,12], but a third generation TKI ponatinib reaches the T315I mutants [4]. Despite the progress in TKI treatment, the disease cannot be considered as cured, since the therapy cannot be stopped in over half of the patients [2,3,13]. Given this data, in 2020, the European Leukemia Net (ELN) recommended that treatment-free remission (TFR) should be a new goal for the CML research [14].

After the initial response to TKIs, 20–25% of CML patients develop secondary resistance involving processes dependent and independent from BCR-ABL1 kinase [15,16]. BCR-ABL-dependent mechanisms of resistance fail to explain 20–40% of resistant cases [17,18]. Therefore, novel clinical approaches in CML should consider BCR-ABL-independent mechanisms of drug resistance.

One of the BCR-ABL-independent mechanisms relies on the altered expression of genes crucial for drug influx or efflux [19], such as P-glycoprotein (PgP, multidrug resistance 1, MDR1), which reduces drug concentration levels and residence time within the cell [20,21]. MDR1 polymorphisms may affect the resistance to imatinib in CML patients [22]. Upregulation of MDR1 causes cross-resistance to many anticancer agents and thus could impact other available therapy options [23]. Therefore, researchers endeavor to develop novel pharmacological approaches to prevent or to overcome MDR1 in CML [24,25].

Leukemia Stem Cells (LSCs), also named leukemia initiating cells, constitute another BCR-ABL1-independent resistance to TKIs responsible for lack of TFR [26,27,28,29]. CML LSCs are a population of rare cells with high BCR-ABL1-independent survival skills that initiate leukemia and contribute to its progression and drug resistance. In result, CML may not be curable with TKIs [30]. Therefore, targeting LSCs has become the main aim in therapeutic approaches to CML, and several strategies have been proposed and explored for achieving TFR [31,32].

To address the still-unmet needs in CML therapy, we synthesized new derivatives of dicarboximides, a group of compounds of long known anticancer activity. Novel compounds exhibited high cytotoxic activity against leukemia cells and low toxicity against normal cells [33]. In particular, one of the derivatives denoted BK124.1 (4-[2-hydroxy-3-(propan-2-ylamino)propyl]-1,7-diethyl-8,9-diphenyl-4-azatricyclo [5.2.1.02,6]dec-8-ene-3,5,10-trione; C_31_H_37_CIN_2_O_4_; derivative 11a in [34]; derivative no 5 in [35]) was selectively toxic towards human leukemia cells such as chronic myelogenous (CML) K562 cells from blast crisis while non-toxic to normal HUVEC [34,35]. Here, we show BK124.1 efficacy against the human CML cells in a xenotransplantation mouse model and in the two groups of particularly resistant cells: K562 cells expressing MDR1 (K562-MDR1) and in CD34^+^/CD38- leukemia stem cells from peripheral blood of CML chronic phase patients.

## 2. Materials and Methods

### 2.1. Reagents

BK124.1 compound was synthesized by us, as described earlier [34]. For all in vitro experiments, 10 mM BK124.1 stock solution was prepared in DMSO and stored light-protected at −20 °C. The final concentration of DMSO in the experiments was 0.1% (v_DMSO_/v_medium_) unless otherwise stated. For in vivo experiments, BK124.1 was dissolved in 10% Solutol HS 15/10% ethanol (Supplementary Methods). Vincristine was purchased from Trimen Chemicals (Łódź, Poland), and all other chemicals from Sigma-Aldrich (St. Louis, MO, USA), unless otherwise stated.

### 2.2. K562 and K562-MDR1 Cell Lines

CML cell line K562 CCL-243™ (BCR-ABL^+^) was purchased from the ATCC^®^ (Manassas, VA, USA). Cells were grown in RPMI 1640 medium containing HEPES (Biowest, Nuaillé, France) with 2 mM GlutaMAX (Gibco, Grand Island, NY, USA), supplemented with 10% fetal bovine serum (Sigma-Aldrich, St. Louis, MO, USA) and 100 µg/mL penicillin and 100 U/mL streptomycin (Gibco, Grand Island, NY, USA) and 0.1 µM Vincristine (for K562-MDR1).

#### Generation of K562-MDR1 Cell Line

The K562-MDR1 cell line was developed using increasing concentrations of the commonly used antimitotic drug vincristine on a polyclonal K562 population, as described earlier [36]. In brief, K562 cells were grown at increasing concentrations of vincristine, starting with 3 nM concentration corresponding to the IC50 value for K562 determined by the MTT assay. The DMSO concentration in the culture was 0.1%. The vincristine dose was doubled every 2–3 weeks (at least twice for each experiment) after the cells reached confluence. Cell’s viability was above 90%, as confirmed by Muse Cell Analyzer (Merck Millipore, Burlington, MA, USA). The final concentration of vincristine reached was 0.1 μM. The increase in the K562-MDR1 population was confirmed by flow cytometry and PCR.

### 2.3. Peripheral Blood Collection and Isolation of CD34^+^ Cells from CML Patients

Blood samples were taken (venipuncture, EDTA-K2 Vacutainer tubes, Becton Dickinson, Franklin Lakes, NJ, USA) from 8 chronic phase CML patients (CML1–CML8; 4 men and 4 women, aged 39–79) diagnosed at the Department of Hematology, University Clinical Center, Medical University of Warsaw, following their written consent. The diagnosis was carried out by qualified clinical staff based on an interview, blood parameters and molecular tests detecting BCR-ABL1 (transcript b3a2). Peripheral blood mononuclear cells were immediately isolated from the fresh blood sample using standard protocol (Supplementary Methods). CD34^+^ cells were isolated with EasySep™ Human CD34 Positive Selection Kit II (StemCell Technologies, Vancouver, BC, Canada) and cultured following kit’s manufacturer protocol. For experiments, cells were always pre-cultured for 24 h.

### 2.4. Measurement of Purity and Apoptosis in CD34^+^/CD38^−^ Cells

Pre-cultured cells were treated with BK124.1 or DMSO for 24 h. For purity analysis, cells were stained with anti-human CD34-APC antibody, anti-human CD38-PE (Biolegend, San Diego, CA, USA), and propidium iodide according to the manufacturers’ protocols. For apoptosis analysis, cells were additionally stained with Annexin V-FITC kit (BD, Franklin Lakes, NJ, USA) according to the manufacturer’s protocol. Appropriate isotype controls were included. Stained cells were immediately analyzed on a BD LSRFortessa flow cytometer with FlowJo software (BD, Franklin Lakes, NJ, USA), after exclusion of doublets. Purity was analyzed on live cells only, and apoptosis separately for each population of cells (CD34^+^/CD38^+^ or CD34^+^/CD38^−^).

### 2.5. Cell Viability Test

Cells were seeded in 96-well plates; after 24 h, medium with the appropriate drug concentration was added to each well. After 48 h, Tetrazolium Bromide (Sigma-Aldrich, St. Louis, MO, USA) was added to each well for 2–3 h. After that, cells were lysed and formazan crystals were dissolved. Absorbance was read using an iMark Microplate absorbance reader (Bio-Rad, Hercules, CA, USA). Results were calculated relative to cells treated with DMSO only.

### 2.6. Apoptosis Measurement

Cells were seeded in 6-well plates (K562 or K562-MDR1) or in 24-well plates (CD34^+^ cells). After 24 h, BK124.1 or DMSO was added. After a further 24 h, cells were stained using the Annexin V-FITC kit for the detection of apoptosis (BD, Franklin Lakes, NJ, USA) and immediately analyzed on a BD FACSCalibur using CellQuest software (BD, Franklin Lakes, NJ, USA).

### 2.7. K562 Cell Cycle Analysis

BK124.1 or DMSO was added to cells and after 24 or 48 h incubation. Cells were fixed and stained with 50 µg/mL propidium iodide (Sigma-Aldrich, St. Louis, MO, USA) with 50 µg/mL DNAse-free RNase. The cell cycle was analyzed on a FACSCalibur cytometer (Becton Dickinson, Franklin Lakes, NJ, USA) using ModFit LT 3.2 software (Verity Software House, Topsham, ME, USA).

### 2.8. P-glycoprotein Analysis

BK124.1 or DMSO were added to cells and after 24 h incubation cells were harvested and stained with FITC Mouse Anti-Human P-glycoprotein (CD243) clone 17F9 (BD, Franklin Lakes, NJ, USA) or an appropriate isotype control. Propidium iodide was added to exclude alive from dead cells. Cells were analyzed on a BD FACSCalibur flow cytometer using CellQuest software (BD, Franklin Lakes, NJ, USA).

### 2.9. Immunoblotting

K562 cells were plated at a concentration of 1–2 × 10^5^ cells/mL in a 6-well plate. After 24 h of pre-incubation, BK124.1 or DMSO was added and cells were incubated for a specified time, after which cells were lysed with RIPA buffer (Sigma-Aldrich, St. Louis, MO, USA) and supplemented with c0mplete Protease Inhibitor (Roche, Basel, Switzerland) and PhosSTOP (Roche, Basel, Switzerland). Protein concentration was measured using Pierce BCA Protein Assay Kit (Thermo Fisher Scientific, Waltham, MA, USA) and adjusted to contain equal amount of protein (20 µg) in equal volume. Protein lysates were separated by SDS-PAGE electrophoresis (TGX StainFree, Bio-Rad, Hercules, CA, USA) and transferred onto the PVDF membranes (AppliChem GmbH ITW Reagents, Darmstadt, Germany). Membranes were blocked with 5% non-fat powdered milk, then incubated with primary antibodies overnight at 4 °C followed by HRP-conjugated antibodies (Supplementary Methods). Blots were developed using Clarity Western ECL Substrate and analyzed with ChemiDoc XRS+ (Bio-Rad, Hercules, CA, USA). Protein levels normalized to controls were determined using ImageJ software.

### 2.10. Cytoplasmic and Nuclear Fraction Isolation

K562 cells were plated at a concentration of 1.5 × 10^5^ cells/mL in a 6-well plate. After 24 h pre-incubation, BK124.1 in DMSO was added and incubated for the specified time. Next, cytosolic and nuclear cell fractions were separated as described [37]. Samples for immunoblotting contained the same amount of protein (at least 15 µg) in the same final volume. The purity of the separated fractions was checked by the level of control proteins: ACTB (beta-actin)—cytoplasmic fraction marker (Cell Signaling Technology, Danvers, MA, USA), LMNB1 (laminin B1)—nuclear fraction marker (Santa-Cruz Biotechnology, Dallas, TX, USA). SDS-PAGE and immunoblotting were performed as described for whole cell lysates.

### 2.11. Animals

Immunodeficient female mice Balb/cOlaHsd-Foxn1nu (Balb/c nude mice, unable to produce T lymphocytes) and NOD.Cg-Prkdcscid Il2rgtm1Wjl/SzJ (NSG mice, unable to produce T and B lymphocytes) were purchased, respectively, from Janvier laboratories (France) and Jackson Laboratory (Bar Harbor, ME, USA). The animals were kept in separate, individually ventilated cages (IVC), with ad libitum access to food and water.

### 2.12. Pharmacokinetics and Toxicity Assays

Five Wistar rats were injected intravenously (IV) with BK124.1 at 5 mg/kg or 10 mg/kg concentration, in 10% Solutol HS15/10% ethanol. The blood was taken from each animal after 5, 15, and 30 min, 1, 2, 6, and 24 h post-injection. Blood BK124.1 concentration was measured using mass spectrometry.

Eight-week-old female Balb/c nude mice were randomly divided into test groups and injected with BK124.1 either intraperitoneally, at 20 mg/kg or 40 mg/kg concentrations, or intravenously at 20 mg/kg concentration. The compound blood concentration was measured 15 min, 6, 12, and 24 h post-injection. For each time point, 4 mice were sacrificed. BK124.1 concentration in the bloodstream was analyzed using mass spectrometry. Toxicology was assessed by examining physiological and morphological changes at the injection site, the appearance of internal organs (liver, heart, kidneys, and spleen), blood tests, body mass, and animal behavior.

### 2.13. Experiments in Xenogeneic Mouse Model

Eight-week-old female NOD.Cg-Prkdcscid Il2rgtm1Wjl/SzJ NSG mice (Jackson Laboratory, Bar Harbor, ME, USA) were injected subcutaneously into the dorsal fold with 1 × 10^6^ K562 cells of the human chronic myeloid leukemia (CML) line in a final volume of 100 µL (0.5% of the mouse weight). On day 3 after cell administration, mice were randomly assigned to each group, and on days 4–17 of the experiment, the selected tested compounds and control solutions were administered intraperitoneally or orally. During the experiment, subcutaneous tumors and body weight were measured daily. Tumor growth during the experiment was measured with an electronic display caliper. The theoretical tumor volume [mm], was calculated according to the formula (a^2^ × b) * 0.5, where a is the shorter tumor axis [mm] and b is the longer tumor axis [mm]. On day 26, the experiment was terminated: blood was collected and analyzed for cellular morphology and standard biochemistry metabolites. Tumors were weighed.

### 2.14. Statistics

Statistical analysis was performed using GraphPad Prism version 9.1.0. All in vitro experiments were performed in at least triplicate biological replicates and statistical results are expressed as mean ± standard deviation (SD). Statistical data of animal experiments are expressed as mean ± standard error of the mean (SEM). For experiments involving patients, the normality of the distribution was checked using the Shapiro–Wilk test and the statistical analysis was performed using one-way ANOVA with Dunnett’s post-test * *p* < 0.05, ** 0.05 > *p* > 0.001, *** 0.001 > *p* > 0.0001, **** *p* < 0.0001. Tumor Growth Inhibition (TGI) was assessed as the percent inhibition of tumor growth for the individual experimental variants, according to the formula: TGI = 100 – (mean tumor volume in treatment group/mean tumor volume under control × 100 TGI (%)). The value calculated in this way shows by what percentage the tumors obtained in a given variant are smaller than the tumors observed for the control group. For Figure 5A,B, dashed line indicates the mean log_10_(BK124.1 concentration) and the grayed area shows the 95% confidence interval calculated by bootstrap resampling of the individual rat measurements at each timepoint.

## 3. Results

**Figure 1:**
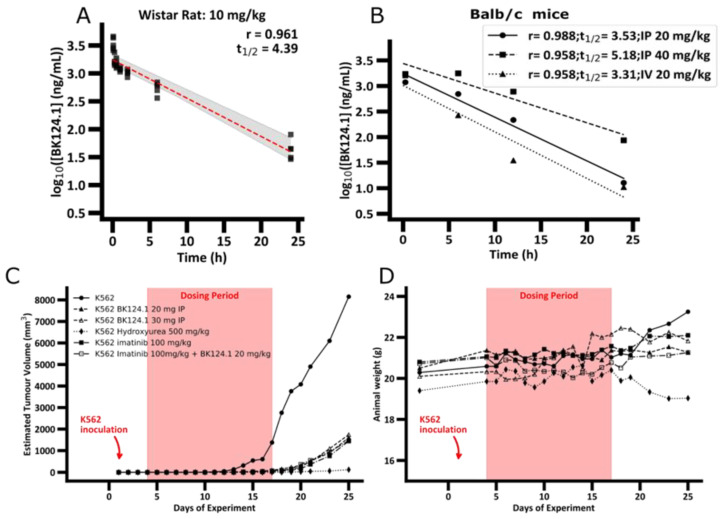
BK124.1 shows pharmacokinetics adequate for chemotherapy and inhibits growth of CML K562 cells in vivo. (**A**,**B**) Pharmacokinetics of BK124.1 in rats and mice. The concentration of BK124.1 in the blood was measured by mass spectroscopy from samples taken within 15 min, 6 h, 12 h, and 24 h from a single administration of the compound. (**A**) Pharmacokinetics in Wistar rats for intravenous (IV) administration of 10 mg/kg—markers show the values for each rat, dashed line indicates the mean concentration values (log_10_ scale), and grey area shows the 95% confidence interval. Data for each timepoint come from 5 animals. (**B**) Pharmacokinetics of BK124.1 in Balb/c nude mice using varied concentrations (20 and 40 mg/kg) and routes of administration (IP = intraperitoneal; IV = intravenous). Data for each timepoint come from 4 animals. (**C**) Estimated tumor volume in K562 xenotransplant mice model (NSG mice injected with K562 treated with specified drugs at various concentrations compared to untreated control). Data for each timepoint come from at least 6 animals. (**D**) Animal weight before and during treatment with drugs and concentrations specified in (**D**).

Based on our previous results showing high cytotoxicity of BK124.1 towards CML K562 cells [34], we began tests of BK124.1 anti-CML potential in vivo employing the Wistar rat and NSG mouse K562 xenograft models. The first step in the in vivo studies of new compounds is to solubilize the compound in aqueous solvents enabling administration in vivo. Acceptable solubility of BK124.1 at the synthesis stage was found only for 100% DMSO [34]. Among a number of solvents tested (Supplementary Table S1), we found that BK124.1 can be stable dissolved in a non-toxic mixture of 10% Solutol HS 15/10% ethanol in water or 0.9% NaCl (see Supplementary Methods). Once the BK124.1 formulation was established, we determined the best route of BK124.1 administration, pharmacokinetics (PK), and non-toxic range of concentration for further in vivo tests of anti-CML activity (Figure 1).

The PK profile of BK124.1 (Figure 1A and Supplementary Table S2) demonstrates that BK124.1 remains detectable in the peripheral blood for 24 h, with steadily dropping concentration (half-life of 4 h 23 min). Follow up experiments with Balb/c nude mice showed that intravenous administration is associated with some signs of local toxicity in the tail. A half-life of BK124.1 administered intraperitoneally (IP) was 3 h 32 min and 5 h 11 min, respectively, for 20 mg/kg and 40 mg/kg (Figure 1B). Since we saw some minor toxic effects in weight, and internal organs’ appearance in mice injected intraperitoneally with 40 mg/kg of BK124.1, but not in mice injected with lower BK124 doses such as 30 mg/kg and 20 mg/kg, we decided to continue testing in vivo with the lower BK124.1 doses.

Next, we investigated the effect of BK124.1 on tumors formed by K562 xenograft in NSG mice. As a control we chose imatinib, as it is the most common first line drug for CML patients, and hydroxyurea as the drug frequently used before the confirmation of the diagnosis to reduce and stabilize blood cell number. The animal experimental groups and the scheme of administration of all tested compounds are presented in Supplementary Table S3. As displayed in Figure 1C,D, prolonged treatment with BK124.1 resulted in a significant reduction in tumor volume, comparable with reduction upon hydroxyurea or imatinib treatments. Figure 1C shows that in the untreated (tumor growth control) mice tumors exhibited exponential growth, starting after 11 days and reaching maximum within the next 14 days, i.e., by 25th day from the inoculation. No statistically significant differences were found between the tumor growth in the intact control animals compared to the vehicle control—animals that were intraperitoneally administered with the BK124.1 solvent (Supplementary Table S4). In contrast to both control groups, tumor growth was effectively inhibited in mice treated with BK124.1 or imatinib, or hydroxyurea (Figure 1C). This pattern is, to some extent, mirrored in the total body weight, and consistent with the increasing tumor volume. In the control animals, the total animal weight showed a large increase in mass in the last 5 days, reaching maximum on day 25 (Figure 1D). The increase in weight in untreated control mice seems to be attributable to the prodigious tumor growth towards the end of the experiment (Figure 1C,D). No such increase occurred in animals treated with BK124.1 or imatinib. Interestingly, the mice dosed with hydroxyurea began to lose large quantities of weight at around day 19, more so than would be expected given the reduction in tumor size, indicating some toxicity of hydroxyurea at this dose (Figure 1D). Moreover, compared to untreated controls, animals treated with BK124.1 or any of the control drugs showed no changes in the morphology or weight of the internal organs including liver, spleen, kidneys, lungs, and heart, and presented blood morphology parameters within the norm (Supplementary Tables S5 and S6).

**Figure 2:**
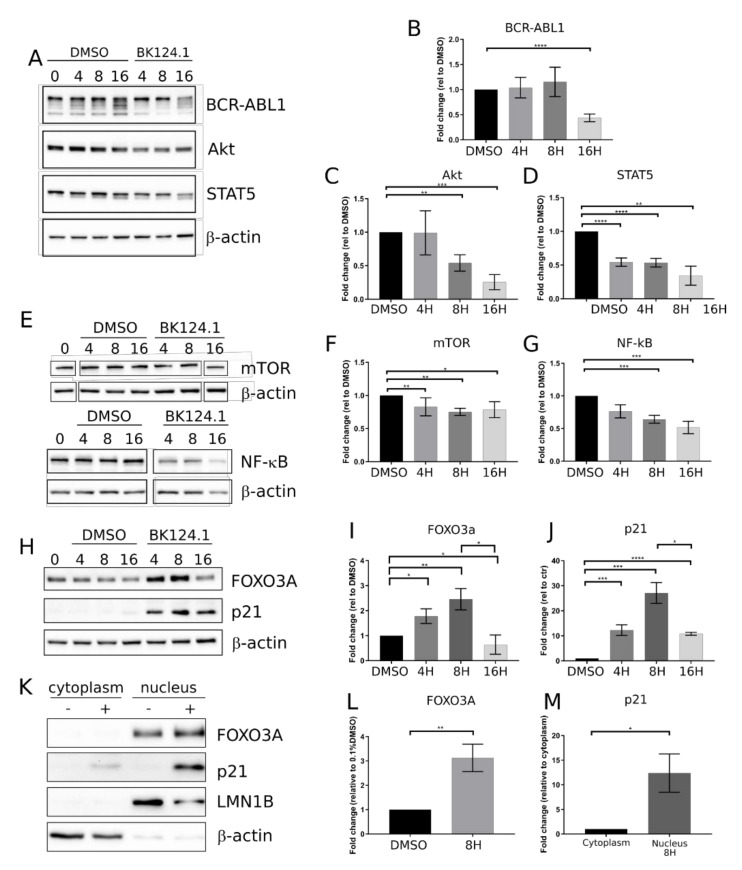
BK124.1 causes a decrease in pro-survival pathways and an increase in proapoptotic proteins. K562 cells were treated for 4, 8, 16, 24, and 48 h with either 0.1% DMSO or BK124.1 compound at a concentration of 2 × IC_50_ (5 µM) in DMSO at 0.1% (v_DMSO_/v_medium_). Protein lysates were collected at 0, 4, 8, and 16 h after treatment and further analyzed by immunoblotting. Representative immunoblots are shown together with densitometry results calculated as a mean fold change in relation to control ± SEM from at least three biological replicates. Statistical analysis was done using a two sided *t* test * *p* < 0.05, ** 0.001 < *p* < 0.05, *** 0.0001 < *p* < 0.001, **** *p* < 0.0001. (**A**–**D**) Cellular levels of BCR-ABL1, Akt, and STAT5. (**E**–**G**) Cellular levels of mTOR and NF-κB. (**H**–**J**) Cellular levels of p21 and FOXO3a. (**K**–**M**) Subcellular localization of p21 and FOXO3a in K562 cells after 8 h of incubation with 5 µM BK124.1. An increase of FOXO3a in nuclear fraction in relation to DMSO treated control (**L**) and an increase of p21 in nuclear protein fraction in relation to cytoplasmic fraction (**M**) are detected.

After confirming anti-CML potential of BK124.1 in the K562 xenotransplant mice model, we investigated the molecular mechanism of BK124.1 activity in K562 cells. We focused on the best known pathways in CML involving the activation of BCR-ABL1 kinase and downstream signaling such as JAK/STAT5, PI3K/AKT, and mTOR/NF-κB [38]. Figure 2 shows the effect of BK124.1 on the levels of the proteins involved in the CML signaling. The first changes following treatment with BK124.1 were observed after 4 and 8 h (Figure 2). A decrease in the level of pro-survival and proliferation stimulating proteins such as BCR-ABL1, STAT5, and AKT (Figure 2A–D) as well as mTOR and NF-κB (Figure 2E–G), with a simultaneous increase in the level of proteins related to apoptosis FOXO3A and p21^waf1/cip1^, were detected (Figure 2 H–J). In fact, p21 in cancer cells can act as an oncogene or tumor suppressor; located in the nucleus it can induce apoptosis, whereas located in the cytoplasm it can exert an anti-apoptotic function [39]. FOXO3A is a transcription factor that can play a role in the initiation of p21 transcription. An increase in the level of FOXO3A was observed after 4 h of incubation with BK124.1, rising to a maximum after 8 h (Figure 2H,I). This increase in the level of FOXO3A was mirrored by an increase in the level of the p21 (Figure 2H,J). The increase in the levels of FOXO3A and p21 proteins takes place mainly in the nuclear fraction of K562 cells after 8 h treatment with 5 µM BK124.1 (Figure 2K–M). Thus, during incubation with the BK124.1, the cytoplasmic synthesis of the p21 protein increases in cells, which then, passing from the cytoplasm to the nucleus, stimulates the process of apoptosis. The results suggest that the mechanism of BK124.1 activity is double-edged, promoting apoptosis and at the same time inhibiting proliferation signaling.

**Figure 3:**
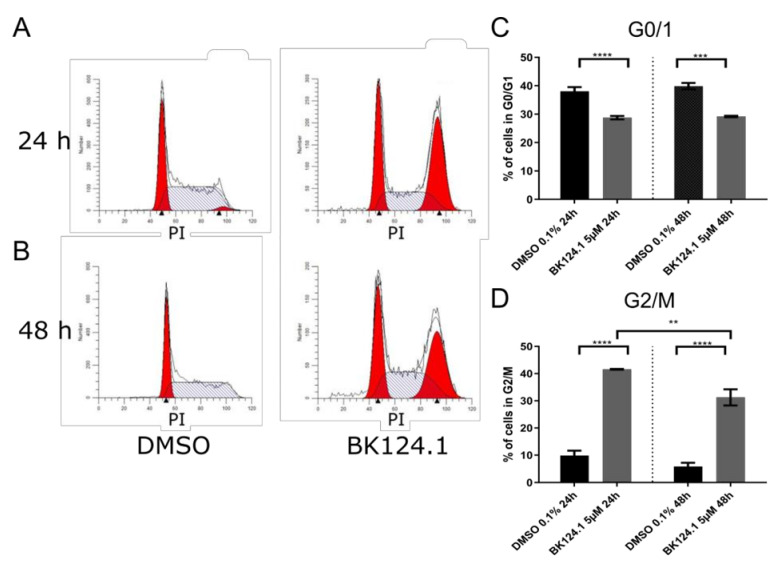
BK124.1 treatment in K562 cells results in G2/M cell cycle phase arrest. Representative flow cytometry histograms show numbers of K562 cells in a particular phase of cell cycle after treatment for 24 h (**A**) or 48 h (**B**) with either 0.1% DMSO (v_DMSO_/v_medium_) or BK124.1 at 5 µM concentration in 0.1% DMSO (v_DMSO_/v_medium_). (**C**,**D**) show mean ± SEM percentage of cells in G0/G1 (**C**) and G2/M (**D**) phase of the cell cycle from at least 3 independent replicates. Statistical analysis was done using 1-way ANOVA test with Tukey’s posttest *p* < 0.05, ** 0.001 < *p* < 0.05, *** 0.0001 < *p* < 0.001, **** *p* < 0.0001.

Consistent with the downregulation of pro-survival and proliferation signaling pathways, we observed a profound G2/M arrest in the cell cycle in K562 cells after 24 h which is emulated after 48 h exposure, but to a lesser extent (Figure 3).

**Figure 4:**
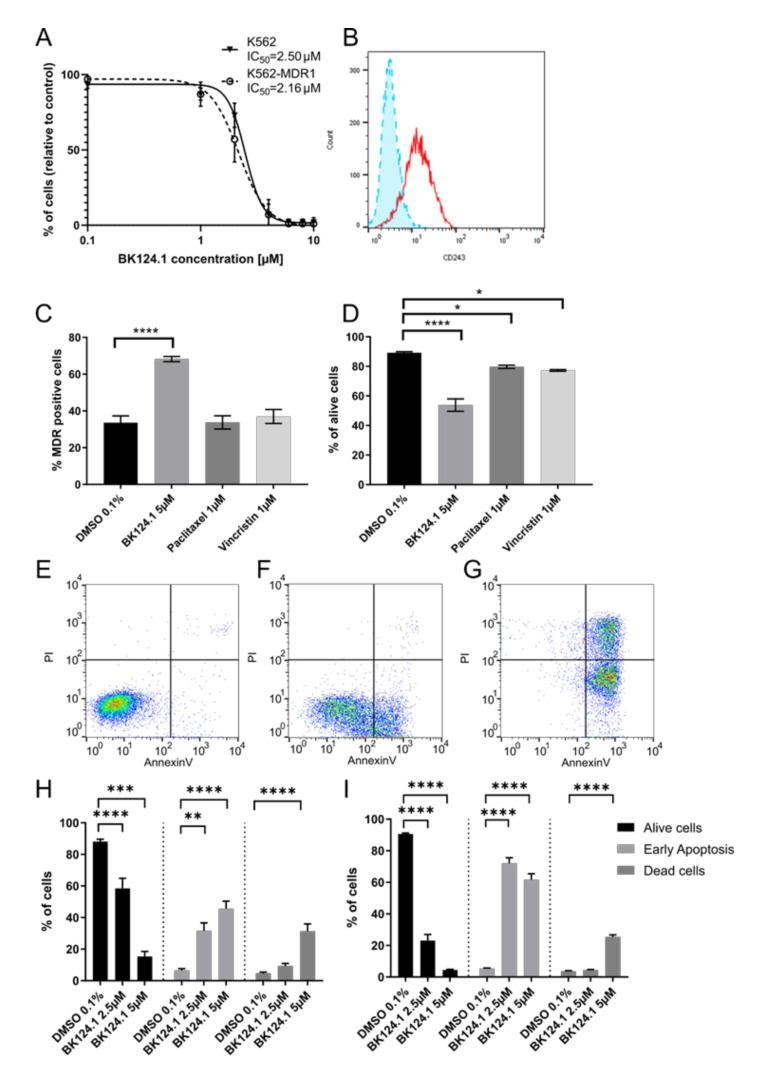
BK124.1 treatment induces apoptosis in K562 and K562-MDR1 cells**.** (**A**) MTT viability test results for K562 and K562-MDR1 cell lines incubated for 48 h in BK124.1 concentrations ranging from 0.1–10 µM. Results show mean (± SD) ratio of absorbance in wells with the compound in relation to absorbance of control wells treated with DMSO from at least 3 biological replicates. (**B**) Flow cytometry histogram showing P-glycoprotein levels detected with FITC mouse anti-human P-glycoprotein antibody (CD243) in K562 (blue, dashed line, filled) and K562-MDR1 (red, solid line) cells. (**C**,**D**). Proportion of K562-MDR1 cells (**C**) and living cells (**D**) following treatment with 0.1% DMSO, 5 µM BK124.1, 1 µM Paclitaxel or 1 µM Vincristine. Data shown as mean percentage ± SEM from at least 3 biological replicates, each in technical duplicate. Statistical analysis done using 1-way ANOVA with Dunnett’s posttest * *p* < 0.05, ** 0.001 < *p* < 0.05, *** 0.0001 < *p* < 0.001, **** *p* < 0.0001. (**E**–**G**), Representative flow cytometry plot showing percentage of K562-MDR1 cells in particular phase of apoptosis as a result of Annexin V-FITC/ Propidium Iodide staining. (**H**–**I**), graphs presenting mean percentage ± SEM of alive cells, cells in early apoptosis or dead cells in K562-MDR1 (**H**) or K562 (**I**) cell lines respectively treated for 24 h with either 0.1% DMSO or BK124.1 in 2.5 µM or 5 µM concentration (in 0.1% DMSO) from at least 3 biological replicates, each in technical duplicate. Statistical analysis was done using 1-way ANOVA with Dunnett’s posttest * *p* < 0.05, ** 0.001 < *p* < 0.05, *** 0.0001 < *p* < 0.00, **** *p* < 0.0001.

As G2/M arrest can lead to cell death by activation of apoptosis or other cell death pathways, we investigated the nature of the cell death induced by BK124.1 in K562 (Figure 4). In addition, we checked if BK124.1 can evoke apoptotic cell death in CML cells with BCR-ABL-independent multidrug resistance MDR1. To investigate if active and robust expression of MDR1 would have an effect on K562 sensitivity to BK124.1 compound, we generated a K562 MDR1 cell line originating from K562 cells (see Supplementary Methods). Following exposure to varied concentrations of BK124.1, we measured the viability of the generated K562-MDR1 cells with an MTT assay (Figure 4A). We found a negligible difference between IC_50_ values for the K562 wild-type and MDR1 variants (Figure 4A) despite clear expression of P-glycoprotein in the K562-MDR1 population but not in control K562 cells (Figure 4B). This result indicates that the MDR1 expression acquired in K562 cells was not sufficient for the cells to escape the cytotoxic effects of BK124.1.

Moreover, Figure 4A–D demonstrates that activity of BK124.1 is clearly different from the activity of the two known antimitotic drugs and MDR1 substrates, paclitaxel, or vincristine, since only BK124.1 overcomes MDR1 in K562 cells. Figure 4D shows that vincristine and paclitaxel reduced cell viability only slightly confirming the resistant phenotype of those cells (mean values 79.7% and 77.2%, respectively, compared to 89.2% in DMSO control), whereas there was a significant drop in viability of K562-MDR1 cells treated with BK124.1 (mean value 53.8%). Figure 4C shows that whilst BK124.1 treatment resulted in almost twice the proportion of K562-MDR1 cells compared with DMSO-treated control (mean value 68.3% compared to 33.6%), neither paclitaxel nor vincristine increased the proportion of K562-MDR1 cells (mean values 33.7% and 37.0% respectively. Importantly, unlike paclitaxel or vincristine, BK124.1 was not able to induce MDR1 in standard K562 cells (Supplementary Figure S1). In this light, an increased proportion of MDR1 cells upon BK124.1 in Figure 4C may not come from MDR1 induction but from other mechanisms, such as differences in cell death kinetics. Furthermore, cross-resistance of K562-MDR1 cells for doxorubicin and paclitaxel was confirmed with an MTT assay (Supplementary Table S7). Altogether, these data indicate that BK124.1 is not a MDR1 substrate and causes massive cell death in the presence of MDR1. The specific effect of BK124.1 on viability of K562-MDR1 cells was determined using a combination of anti-CD243 antibody and propidium iodide. Examination of the cytotoxicity of these compounds against K562-MDR1 cells showed the overall potency only of BK124.1 for breaking the multidrug resistance.

As demonstrated in Figure 4E–I, both 2.5 and 5 µM concentrations of BK124.1 significantly increased apoptosis in K562 cells (72.1% and 61.2% respectively) and in K562-MDR1 cells (60.5% and 30.1% respectively). These data suggest that both cell lines die by apoptosis and thus that the mechanism enabling the MDR1 is not contributing to any apoptosis-escape function. Given the increase in K562 cells in G2/M phase of the cell cycle observed from 24–48 h exposure to BK124.1 (Figure 3), the BK124.1 cytotoxicity is most probably due to apoptosis of replication-arrested cells. Increased MDR1 expression does not affect K562 cells’ sensitivity to BK124.1.

**Figure 5:**
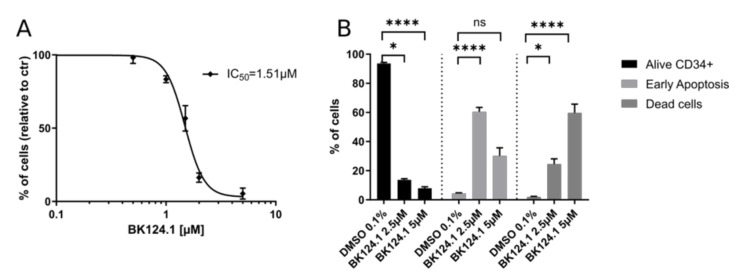
BK124.1 is highly cytotoxic and triggers apoptosis in human CML CD34+ stem and progenitor cells. CD34+ cells were isolated by immunomagnetic separation from peripheral blood mononuclear cells (PBMC) derived from 5 newly diagnosed CML patients at chronic phase. After isolation, CD34+ cells were cultured for 24 or 48 h in the presence of increasing concentrations of BK124.1 and then their viability and apoptotic response were analyzed by MTT and annexin V flow cytometry assays. (**A**) Results of MTT viability assay for CML CD34+. The graph shows the mean ± SD cell viability after 48 h at each BK124.1 concentration for five patients except at 5 µM where n = 3. (**B**) Results of the Annexin V apoptosis assay for CML CD34+ cells after 24 h incubation with 2.5 µM or 5 µM BK124.1 compared to 0.1% DMSO control. The experiment was performed in at least 3 biological replications, each with a technical duplicate. Normal distribution was checked using the Shapiro–Wilk test, statistical analysis was performed using one-way ANOVA with Dunnett’s post-test * *p* < 0.05, **** *p* < 0.0001.

Next, we investigated if BK124.1 induces apoptosis in another type of CML cell population particularly resistant to chemotherapy, i.e., in the CML CD34^+^ stem and progenitor cells from patients (Figure 5). The BK124.1 proved highly cytotoxic to CD34^+^ cells; the IC_50_ value was even slightly lower than observed with K562 cells (Figure 5A). Consistently with the determined IC_50_, CD34^+^ cells treated with either 2.5 or 5 µM BK124.1 showed pronounced apoptotic cell death with viability measured at 10% and 5%, respectively, and in a dose-dependent manner. Interestingly, the early apoptotic response was elevated for the lower concentration of BK124.1 (Figure 5B).

**Figure 6:**
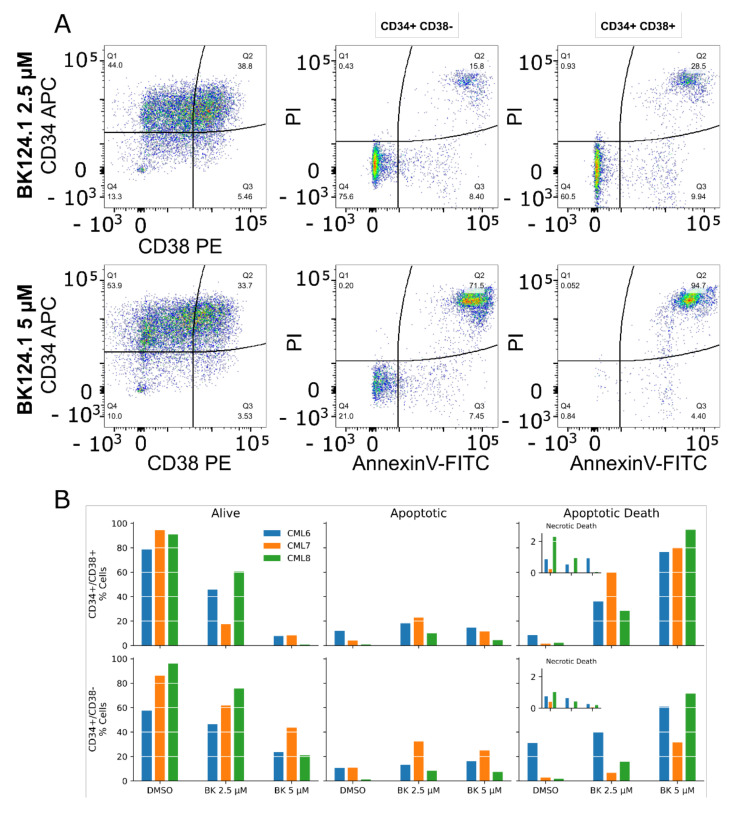
BK124.1 causes apoptotic death in CD34^+^/CD38^−^ leukemic stem cells from CML patients. Results of apoptosis assessment with Annexin V-FITC/Propidium iodide flow cytometry assay in CD34^+^/CD38^+^ and CD34^+^/CD38^−^ cell subpopulations from CML patients after treatment for 24 h with 0.1% DMSO or BK124.1 at the concentration of 2.5 µM or 5 µM. (**A**) Representative flow cytometry images. (**B**) Percentages of cells in subpopulations identified based on CD34/CD38 antigens and Annexin V-FITC/PI staining (alive, early apoptotic, apoptotic death). Data from individual patients are presented.

We deepened the analysis of the apoptotic death triggered by BK124.1 into the subpopulations of CML CD34^+^ cells distinguished by double labeling with anti-CD34 and anti-CD38 antibodies, particularly focusing on the CML CD34^+^/38^−^ cells corresponding to leukemia stem cells (Figure 6). The analysis involved CD34^+^/CD38^−^ cells from 3 newly diagnosed CML patients after checking the percentages of cells in each CD34/CD38 subpopulation (purity) before and following treatment with BK124.1. Already within 24 h, BK124.1 induced apoptosis in CD34^+^/CD38^−^ leukemia stem cells from all the patients. The apoptotic death of CD34^+^/CD38^−^ LSC in response to BK124.1 was dose dependent and higher at 5 µM BK124.1 concentration. As expected, CD34^+^/CD38^+^ cells with lower stem cell potential and resistance responded with even higher apoptosis to BK124.1. The individual differences among patients probably reflect the variability of molecular mechanisms of LSC chemoresistance in various patients.

## 4. Discussion

After the success of TKIs in extending the lives of CML patients, now the main challenge is elimination of CML cells resistant to TKIs, particularly leukemic stem cells, and reaching treatment-free remission [29,40]. Several therapeutic strategies have been suggested [41,42,43], but these investigations are at an early stage. The search is ongoing for non-toxic therapeutics that overcome the mechanisms of drug resistance and the two main dysregulated processes in CML cells: uncontrolled proliferation and blocked apoptosis.

Dicarboximides, as a large class of compounds, have been tested and used as drugs for many pathologies, including cancer. The best known phthalimide derivative, thalidomide, is now used in the treatment of multiple myeloma and myelodysplastic syndrome. Unfortunately, in most tumors, dicarboximides showed high toxicity and were not approved for clinical use [44,45]. Therefore, there have been multiple attempts by others and us to obtain dicarboximide derivatives with lower toxicity [34,35,46,47]. Among 39 newly synthesized, we identified BK124.1 as one of the most potent antileukemic compounds in vitro, showing low toxicity towards normal human endothelial cells [34,35].

Here, we demonstrate that BK124.1 shows anti-CML efficacy in vivo in non-toxic range of concentrations. The advantages of BK124.1 as a potential chemotherapeutic are also its low molecular weight, pharmacokinetics parameters, and the low toxicity of combined therapy of BK124.1 with imatinib. A unique feature of BK124.1 is its high cytotoxicity to CML cells resistant to chemotherapy due to BCR-ABL1-independent mechanisms, such as K562-MDR1 cells with elevated PgP efflux pump or CD34^+^ progenitor and CD34^+^/CD38^−^ stem cells from CML patients. Unlike many commonly used chemotherapeutics such as vincristine, BK124.1 was unable to induce MDR1 expression in K562 cells incubated with low compound concentration for 6 months. These findings indicate that BK124.1 shows potential to overcome at least some BCR-ABL1-independent mechanisms of chemotherapy resistance.

This study demonstrates that BK124.1 also affects BCR-ABL1-dependent signaling cascades. While BK124.1 is not a direct inhibitor of BCR-ABL1 kinase, it influences levels of BCR-ABL1 kinase and its key downstream kinases such as JAK/STAT and PI3K/Akt, known activators of survival and proliferation pathways in CML cells. Lowered levels of the key pro-leukemic kinases in CML K562 blast cells were detected within 4–8 h upon incubation with BK124.1, associated with nuclear translocation and increased levels of proapoptotic proteins FOXO3A and p21. These changes were consistent with their arrest at the G2/M phase of the cell cycle and death due to apoptosis. Thus, this mechanism accounts for the BK124.1 ability to block proliferation and induce apoptosis in CML blasts. As cancer cells escape control of proliferation and apoptosis, the BK124.1 mechanism of activity corresponds well to the assumptions of presently designed anti-CML and more general anti-cancer chemotherapies. The ability of compounds to induce cancer cell death by apoptosis, without a release of cell debris and inflammatory reactions, is particularly advantageous from a therapeutic standpoint.

Lowering the levels of PI3K and Akt can arrest the cell cycle progression and promote induction of apoptosis. In particular, Akt phosphorylates FOXO transcription factors—including FOXO3A—blocking it in the cytoplasm, which prevents its proapoptotic activity [48]. Akt also phosphorylates p21, sequestering it in cytoplasm where p21 is engaged in anti-apoptotic activity [39]. Thus, BK124.1-triggered lowering of Akt levels results in the increase in FOXO3A and p21 level in the nuclear fraction. Nuclear p21 is recognized for its function as an inhibitor of cell cycle kinases (CDK), enabling exquisite control of the cell cycle progression and cell cycle arrest at G1 or G2 phase, and also as a proapoptotic protein. The increased levels of p21 protein in the nucleus of CML cells treated with BK124.1 can explain the observed G2/M arrest and apoptosis of these cells. Thus, our results have revealed an interesting proapoptotic mechanism of BK124.1 in CML cells involving enhanced nuclear translocation of FOXO3a and its target p21.

High levels of nuclear FOXO3a might also explain the observed induction of PgP expression on the cell surface. FOXO3a can bind to the proximal promoter region of the ABCB1 gene and thus regulates MDR1 expression [49]. However, BK124.1 causes apoptosis in CML cells regardless of MDR1 expression and showed similar IC_50_ values against both K562 and K562-MDR1 cells, implying that BK124.1 itself is either a poor substrate for PgP, or is not one at all. This is in direct contrast to the finding that second generation drugs such as dasatinib and nilotinib are seen in vitro to be substrates for the PgP pump [50]. Since it seems that MDR1 does not affect BK124.1 intracellular concentration, it might be worth investigating its effect not only in CML but also in other cancer types exhibiting MDR1. MDR1 expression is often observed in cancer cells exposed to common chemotherapeutics that are substrates for PgP, which results in drug efflux and therapy failure. The attempts to decrease the efflux activity of PgP during chemotherapy remain largely unsuccessful. Proposed explanations are poor efficacy and high toxicity of small molecule inhibitors targeted to MDR1 [51].

We confirmed the anti-leukemic effect of BK124.1 on CD34^+^ cells isolated from CML patients. Moreover, BK124.1 induced apoptosis in the rare CD34^+^/CD38^−^ leukemia initiating stem cells. CML LSCs serve as reservoirs to drive relapse, recurrence, or progression to more aggressive leukemia forms. In order to achieve treatment-free remission, which has become a key evaluation criterion for CML therapies, the search continues for drugs that efficiently eliminate LSCs, cells which are refractory to standard therapies mainly due to differing BCR-ABL1-independent mechanisms [27]. Moreover, the mechanisms of resistance to chemotherapy of LSCs can be patient-specific, which may explain observed differences in sensitivity to BK124.1 of CD34^+^/CD38^−^ cells from individual patients. Given these findings, BK124.1 is promising for development of future novel individualized therapies towards TFR in CML.

The future development of BK124.1 as a chemotherapeutic for treatment of CML should evaluate BK124.1 effects in the hematopoietic stem cells in relation to factors of their natural microenvironment in the bone marrow. These are challenging studies, because the deeper phenotypic characteristics of CML stem cells are complex and methods allowing clear identification of these cells are currently under development. In addition, subsequent experiments should consider the local microenvironment in bone marrow, as it affects stem cells responses to chemotherapeutics and to the achievement of TFR [52]. As such, it is probable that deleterious impacts on LSCs are necessary but not sufficient to limit relapse of CML [53]. Accurate delivery to the appropriate location, as well as understanding the local context of the targeted cells, would be critical for further development of therapeutic strategies directed at leukemia initiating stem cells using BK124.1. Finally, while BK124.1 lowers levels of BCR-ABL1, the toxicity caused by BK124.1 does not seem to be due to BCR-ABL1 kinase inhibition, and so the mutations in BCR-ABL1 itself should not affect CML sensitivity to the compound. However, it would certainly require further experimental evaluation.

## 5. Conclusions

This study provides the first evidence from animal and cellular models that BK124.1 has several unique advantages as a novel potential anti-CML chemotherapeutic, including the ability to evoke massive apoptosis in multidrug resistant CML blasts (MDR1) and in the CD34^+^/CD38^−^ cells from CML patients containing deleterious tumor-initiating leukemia stem cells. As BK124.1 both downregulates BCR-ABL1-dependent signaling and overcomes BCR-ABL1-independent drug resistance mechanisms, future development of BK124.1 could offer curative treatment of CML and of other cancers resistant or intolerant to presently used drugs.

## 6. Patents

Patent application—(date, number): 17.02.2021, P.437038; 17.02.2022 PCT/IB2022/051411. Title: “Dicarboximide derivative for use in the treatment of cancer”. Inventors: Grażyna Hoser, Iga Stukan, Marek Gryzik, Mikołaj Zdioruk, Marcin Cieślak, Mariola Napiórkowska, Karolina Królewska-Golińska, Barbara Nawrot, Urszula Wojda.

## Data Availability

All data generated and analyzed in this study are available from the corresponding author upon justified request.

## Supplementary Materials

The following supporting information can be downloaded at: https://www.mdpi.com/article/10.3390/cancers14153641/s1, Supplementary figures: Table S1: Solubility of BK124.1 in selected solvents. In bold are marked solvents in which BK124.1 is soluble and stable, adequate for the administration in vivo; Table S2: Key pharmacokinetic parameters of BK124.1 in BALBc mice following IV or IP administration at the indicated doses; Table S3: Experimental groups in the testing of BK124.1 efficacy against CML cells in vivo; Table S4: Differences in tumor weights in the testing of BK124.1 efficacy against CML cells in vivo; Table S5: Weight of mouse organs (mean ± SD) after 14-day continuous dosing of compounds (BK124.1, imatinib—IM, hydroxyurea); Table S6: Blood morphology parameters (mean ± SD) in mice after 14-day continuous dosing of compounds (BK124.1, imatinib (IM), hydroxyurea); Table S7: MTT viability test results and calculated IC_50_ for K562 and K562-MDR1 cell lines incubated for 48 h in Vincristine (A), Paclitaxel (B) or Doxorubicine (C); Figure S1: Incubation of K562 cells in low concentration of BK124.1 for 6 months does not result in inducing MDR1 and acquiring resistance. Raw Western Blots: Figure S2: Whole blots for Western blots in Figure 2A, Figure S3: Whole blots for Western blots in Figure 2E (p65); Figure S4: Whole blots for Western blots in Figure 2E mTOR; Figure S5: Whole blots for Western blots in Figure 2H; Figure S6: Whole blots for Western blots in Figure 2K. Densitometry for Figure 2B–D,F,G,I,J,L, M. Supplementary Methods (detailed methodology).
