# Supplementary material for: Novel Dicarboximide BK124.1 Breaks Multidrug Resistance and Shows Anticancer Efficacy in Chronic Myeloid Leukemia Preclinical Models and Patients’ CD34+/CD38− Leukemia Stem Cells
Source: Cancers (Basel). 2022 Jul 27;14(15):3641. doi: 10.3390/cancers14153641 (PMC9332833; doi:10.3390/cancers14153641)
Supplement: Supplementary file 1 [file cancers-14-03641-s001.zip › cancers-1789529-supplementary materials .pdf]

Supplementary Information

Additional file 1:

Table S1. Solubility of BK124.1 in selected solvents. In bold are marked solvents in which BK124.1 is soluble and stable, adequate for the administration in vivo.

Solvent	Formulation	Comments
Water	0.9% NaCl	insoluble
	10% ethanol in 0.9% NaCl	insoluble
Cyclodextrin	α-cyclodextrin in water	insoluble
	β-cyclodextrin in water	insoluble
	γ-cyclodextrin in water	insoluble
DMSO	DMSO 100%	soluble/stable
	DMSO < 100% in water	insoluble
Surfactant	20% Solutol HS 15 in 0.9% NaCl	insoluble
	20% Solutol HS 15 in PBS	insoluble
	**20% Solutol HS 15, 10% ethanol in water 0.9% NaCl**	**soluble/stable**
	**10% Solutol HS 15, 10% ethanol in water 0.9% NaCl**	**soluble/stable**

Table S2. Key pharmacokinetic parameters of BK124.1 in BALBc mice following intravenous (IV) or intraperitoneal (IP) administration at the indicated doses.
C_0: maximum plasma concentration extrapolated to t = 0; $t_{1/2}$: half-life; MRT_{last}: mean residence time, calculated to the last observable time point; CL: clearance; V_{ss}: steady state volume of distribution; AUC_{last}: area under the curve, calculated to the last observable time point; AUC_{∞}: area under the curve, extrapolated to infinity.

[a] Vehicle: 10% Solutol HS 15/10% ethanol in H_2O
[b] Dose normalized by dividing the parameters by the nominal dose in mg/kg

	Whole blood, female Balb c mouse		
	**IV**	**IP**	**IP**
Dose (mg/kg)	20 [a]	20 [a]	40 [a]
C_0 (ng/mL)	1024	1740	2759
$t_{1/2}$ (h)	3.31	3.53	5.18
MRT_{last} (h)	4.78	5.09	7.48
CL (L/h/kg)	0.0604	0.0418	0.0341
V_{ss} (L/kg)	0.391	0.23	0.29
AUC_{last} (h·ng/mL)	50.10	64.86	647.26
AUC_{∞} (h·ng/mL)	6618	9579	23480
AUC_{last}[b] (h·kg·ng/mL/mg)	2.50	3.24	16.18
AUC_{∞}[b] (h·kg·ng/mL/mg)	331	479	587

Table S3. Experimental groups in the testing of BK124.1 efficacy against CML cells in vivo. Control and treated mice were grouped according to the tested compounds and the scheme of their administrations (doses, administration frequencies, routes of administration) in the CML xenogeneic model obtained by subcutaneous transplantation of 1×10^6 cells of the human CML blast line K562 into mice NOD.Cg-Prkdcscid Il2rgtm1Wjl/SzJ (NSG). IP, intraperitoneal administration; O, orally administration.

Group/ treatment	Trans plant	Dose, route of administration	Solvent	Dose frequency	Animal number /group
BK124.1	K562	20 mg/kg, IP	10% Solutol HS 15/ 10% ethanol	1 x daily	8
BK124.1	K562	30 mg/kg, IP	10% Solutol HS 15/ 10% ethanol	1 x daily	8
Imatinib	K562	100 mg/kg, O	no solvent	1 x daily	12
Hydroxyurea	K562	500 mg/kg, IP	Aqua pro injectione	1 x daily	8
BK124.1 +<br>Imatinib	K562	20 mg/kg, IP<br>100 mg/kg, O	10% Solutol HS 15/ 10% ethanol<br>100 mg/kg	1 x daily<br>1 x daily	7
solvent (vehicle control)	K562	400 µl/kg, IP	10% Solutol HS 15 / 10% ethanol	1 x daily	4
-<br>(tumor growth control)	K562	-	-	-	15
-<br>(intact control)	-	-	-	-	4

Table S4. Differences in tumor weights in the testing of BK124.1 efficacy against CML cells in vivo.

No significant differences in tumor weights [mg] at the end of experiment between untreated animals (tumor growth control) and vehicle-treated animals (vehicle control) in the testing of BK124.1 efficacy against CML cells in the xenogeneic mouse model as described in Table S3. The CML xenogeneic mouse model was obtained by subcutaneous transplantation of K562 cells in NSG mice. Vehicle control - animals which were administered intraperitoneally with the 10% Solutol HS 15 / ethanol 10% at the dose of 400 µl/per mouse. The CML xenogeneic mouse model was obtained by subcutaneous transplantation of K562 cells in NSG mice.

	**N**	**Median [mg]**	**P25 [mg]**	**P75 [mg]**	**Mean [mg]**	**SD [mg]**
**Tumor growth control**	4	7.49	6.77	8.20	7.48	1.92
**Vehicle control**	4	7.00	6.29	7.71	7.00	1.66

Table S5 Weight of mouse organs (mean ± SD) after 14-day continuous dosing of compounds (BK124.1, imatinib - IM, hydroxyurea).

Weight of mouse organs (mean ± SD) after 14-day continuous dosing of compounds (BK124.1, imatinib - IM, hydroxyurea) compared to intact and tumor growth control groups in the testing of BK124.1 efficacy against CML cells in the xenogeneic mouse model as described in Table S3. N=4 in each group. Compared to controls, animals treated with BK124.1 or any of the control drugs showed no changes in the weight of the internal organs.

**Parameter**	**Intact control**	**Transplated K562 cells**					
		**Tumor growth control**	**BK.124.1**		**IM**	**BK.124.1 + IM**	**Hydroxyurea**
**Dose (mg/kg)**	-	-	20	30	100	20 + 100	500
**Liver (g)**	1.21±0.13	1.07±0.16	1.09±0.10	1.27±0.11	1.12±0.21	1.12±0.09	1.16±0.13
**Brain (g)**	0.47±0.02	0.46±0.02	0.47±0.02	0.47±0.01	0.47±0.02	0.47±0.01	0.46±0.02
**Kidneys (g)**	0.28±0.01	0.26±0.03	0.26±0.03	0.28±0.02	0.29±0.05	0.29±0.06	0.27±0.04
**Splean (g)**	0.01±0.00	0.01±0.00	0.03±0.02	0.03±0.01	0.02±0.01	0.02±0.01	0.05±0.01
**Lung (g)**	0.24±0.00	0.28±0.08	0.24±0.10	0.34±0.02	0.27±0.06	0.30±0.08	0.25±0.08
**Heart (g)**	0.50±0.57	0.10±0.02	0.10±0.00	0.10±0.02	0.11±0.02	0.12±0.01	0.11±0.03
**Tumor weights (g)**	-	9.2±2.1	1.6±1.6	1.9±1.8	2.5±2.3	2.1±1.6	1.8±0.9

**Mouse weight (g)**	22.5±0.8	25.4±2.7	22.3±0.8	21.8±1.5	22.5±1.8	20.3±1.4	20.3±1.4

Table S6 Blood morphology parameters (mean ± SD) in mice after 14-day continuous dosing of compounds (BK124.1, imatinib (IM), hydroxyurea).

Blood morphology parameters (mean ± SD) in mice after 14-day continuous dosing of compounds (BK124.1, imatinib (IM), hydroxyurea) compared to intact and tumor growth control groups in the testing of BK124.1 efficacy against CML cells in the xenogeneic mouse model as described in Table S3. N=4 in each group. Animals treated with BK124.1 or any of the control drugs presented overall blood morphology parameters within norm.

Abbreviations: WBC: white blood cells; RBC: red blood cells; HGB: hemoglobin; HCT: hematocrit; MCV: mean corpuscular volume; MCH: mean corpuscular hemoglobin; PLT: Platelet Count; MPV: mean platelet volume; platelets; PCT: platelet hematocrit; RDW: red cell distribution width; MCHC: mean corpuscular hemoglobin concentration; MON: monocyte; GRA: granulocyte. Standard range for mouse according to Scil Vet ABC ™Hematology Analyzer (Scil Animal Care Company).

**Parameter** (Standard range)	**Intact Control**	**Transplanted K562 cells**					
		**Tumor growth Control**	**BK.124.1**		**IM**	**BK.124.1 + IM**	**HYDR**
**Dose**	-	-	20 mg/kg	30 mg/kg	100 mg/kg	20 + 100 mg/kg	500 mg/kg
**WBC** (10^3/μL) (3.0-15)	4.7±0.4	17.0±5.2	14.9±4.7	10.5±5.0	4.1±2.4	8.3±5.0	8.1±4.3
**RDW** (%) (11.5-14.5)	12.9±0.2	13.7±0.2	13.1±0.4	12.9±0.1	12.5±0.3	12.7±0.2	17.3±0.6
**RBC** (10^6/μL) (5.0-12)	7.9±0.4	6.9±0.2	7.1±0.4	7.9±0.2	8.3±0.8	8.1±0.5	6.9±0.4
**PLT** (10^3/μL) (140-600)	830±30	1202±19	1658±235	1437±261	1295±277	1619±240	1575.5±606.9
**MPV** ($μm^3$) (4.6-7.3)	5.5±0.1	5.1±0.2	5.0±0.2	5.1±0.2	4.9±0.3	5.0±0.2	3.6±4.3
**MON** (%) (0.0-99.0)	10.0±1.1	16.3±1.9	17.8±2.8	15.4±3.8	17.1±4.1	15.3±1.8	16.0±3.8

**MCV** (µL) (44-69)	55.5±0.7	53.6±0.5	53.3±0.5	52.8±1.0	54.0±0.8	52.5±0.6	60.0±1.7
**MCHC** (g/dL) (21.6-42)	32.5±0.2	32.8±0.4	32.4±0.3	32.4±0.6	31.7±0.6	32.3±0.2	31.5±0.3
**MCH** (pg) (12-24.5)	18.3±0.2	17.6±0.1	17.3±0.3	17.2±0.4	17.1±0.2	16.9±0.3	18.9±0.5
**LYMP** (%) (0.0-99.0)	63.6±4.9	35.48±9.3	42.9±10.9	41.6±18.5	53.6±7.1	58.5±11.0	58.0±9.9
**HGB** (g/dL) (1.1-18)	14.2±0.7	12.2±0.3	12.3±0.7	13.5±0.5	14.1±1.2	53.6±7.1	13.1±0.8
**HCT** (%) (36-52)	43.8±2.0	37.0±1.0	38.0±2.3	41.7±0.9	44.6±4.0	42.6±3.3	41.5±2.4
**GRA** (%) (0.0-99.0)	26.5±3.8	48.3±8.9	39.3±9.8	43.1±15.7	29.3±5.6	26.3±10.1	26.0±7.6

Table S7. MTT viability test results and calculated IC_{50} for K562 and K562-MDR1 cell lines incubated for 48 hours in Vincristine (A), Paclitaxel (B) or Doxorubicine (C). Results show mean (± SD) ratio of absorbance in wells with the compound in relation to the absorbance of control wells treated with DMSO from at least 3 biological replicates.

A)

Vincristine			
	µM	Viability in relation to control	
		K562	K562-MDR1
	0	100,0%	100,0%
	0,001	76,6% ± 2,0%	106,4% ± 8,6%
	0,005	32,6% ± 0,5%	110,9% ± 3,8%
	0,01	24,6% ± 2,5%	105,9% ± 2,4%
	0,1	17,6% ± 0,0%	94,3% ± 11,3%
	1	16,9% ± 2,8%	32,4% ± 9,3%
**IC_{50}**		**0,003 µM**	**0,512 µM**

B)

Paclitaxel			
	µM	Viability in relation to control	
		K562	K562-MDR1
	0	100,0%	100,0%
	0,0005	45,1% ± 12,8%	100,7% ± 5,0%
	0,005	32,6% ± 12,1%	100,5% ± 6,4%
	0,05	23,3% ± 7,3%	89,9% ± 6,9%
	0,5	21,5% ± 6,6%	48,4% ± 6,2%
	5	22,6% ± 5,8%	44,3% ± 5,7%
**IC_{50}**		**0,4 µM**	**7 µM**

C)

Doxorubicin			
	µM	Viability in relation to control	
		K562	K562-MDR1
	1	100,0%	100,0%
	0,005	97,7% ± 5,1%	99,4% ± 5,1%
	0,05	90,4% ± 15,7%	101,1% ± 8,0%
	0,5	35,9% ± 14,1%	84,1% ± 10,3%
	5	13,2% ± 4,5%	45,1% ± 12,8%
**IC_{50}**		**3,7 µM**	**7 µM**

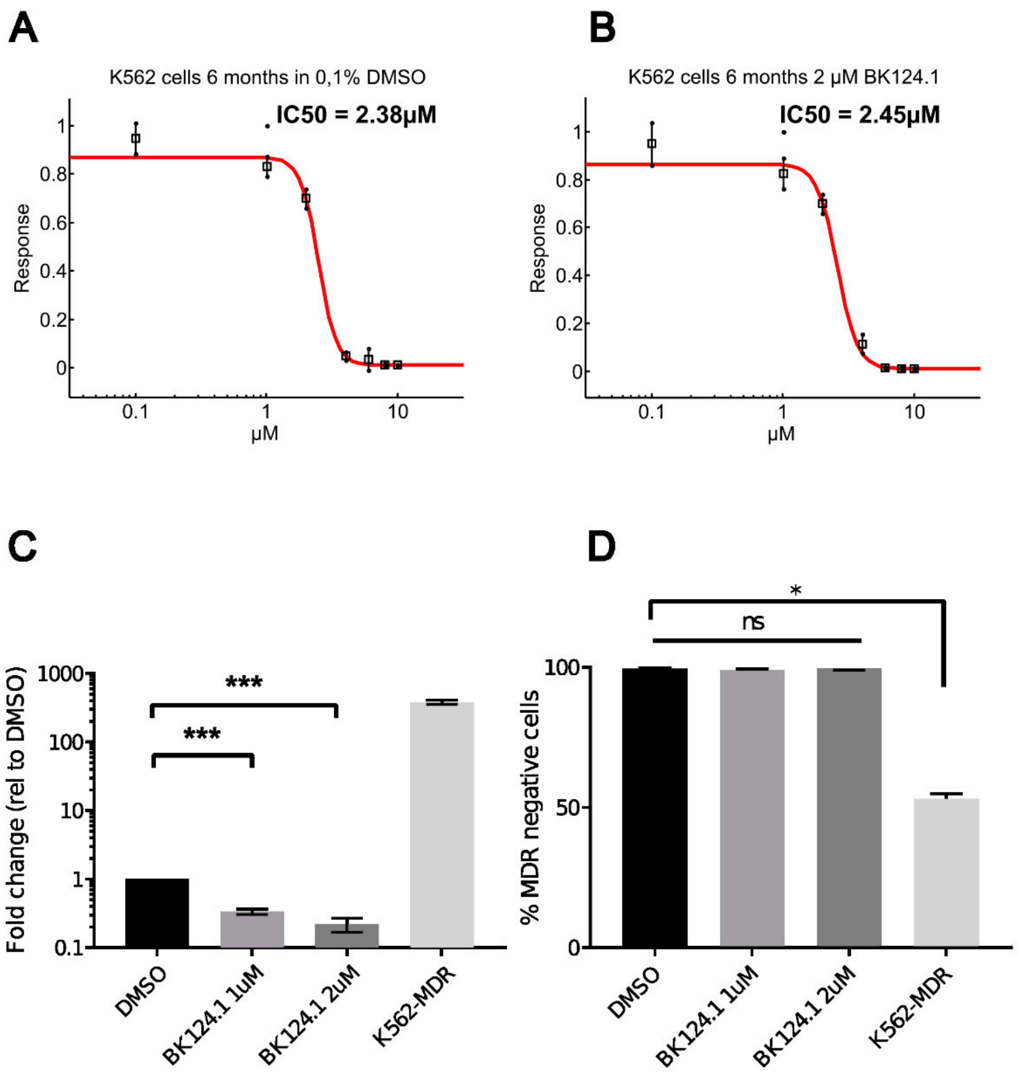

Figure S1. **Incubation of K562 cells in low concentration of BK124.1 for 6 months does not result in inducing MDR1 and acquiring resistance.** K562 cells were cultured in either 0,1% DMSO, 1 µM or 2 µM BK124.1 (v/v) for at least 6 months.

(A-B) MTT viability test result for K562 cells incubated for 48h in BK124.1 concentrations ranging from 0,1 µM -10 µM. Results shown as mean ±SD ratio of absorbance in wells with the compound in relation to absorbance of control wells treated with DMSO from at least 3 independent experiments.
(C) Graph shows relative fold change of ABCB1 mRNA in K562-MDR1 cells or in K562 cells kept in DMSO 0,1%, 1 µM and 2 µM BK124.1 respectively for at least 6 months. Statistical analysis done using 1-way ANOVA with Dunnett's posttest* P<0.05,** 0.001<P<0.05
(D) Graph shows mean±SEM percentage of MDR1-negative cells calculated using flow cytometry. Statistical analysis done using Kruskall-Wallis with Dunn's posttest * P<0.05,** 0.001<P<0.05

Figure S2 Whole blots for Western blots in Figure 2A.

1. BCR/ABL1 (~ 210kDa)

1 – K562 + DMSO T_0
2 – K562 + DMSO 4h
3 – K562 + DMSO 8h
4 - K562 + DMSO 16h
5 – K562 + BK124.1 4h
6 – K562 + BK124.1 8h
7 – K562 + BK124.1 16h

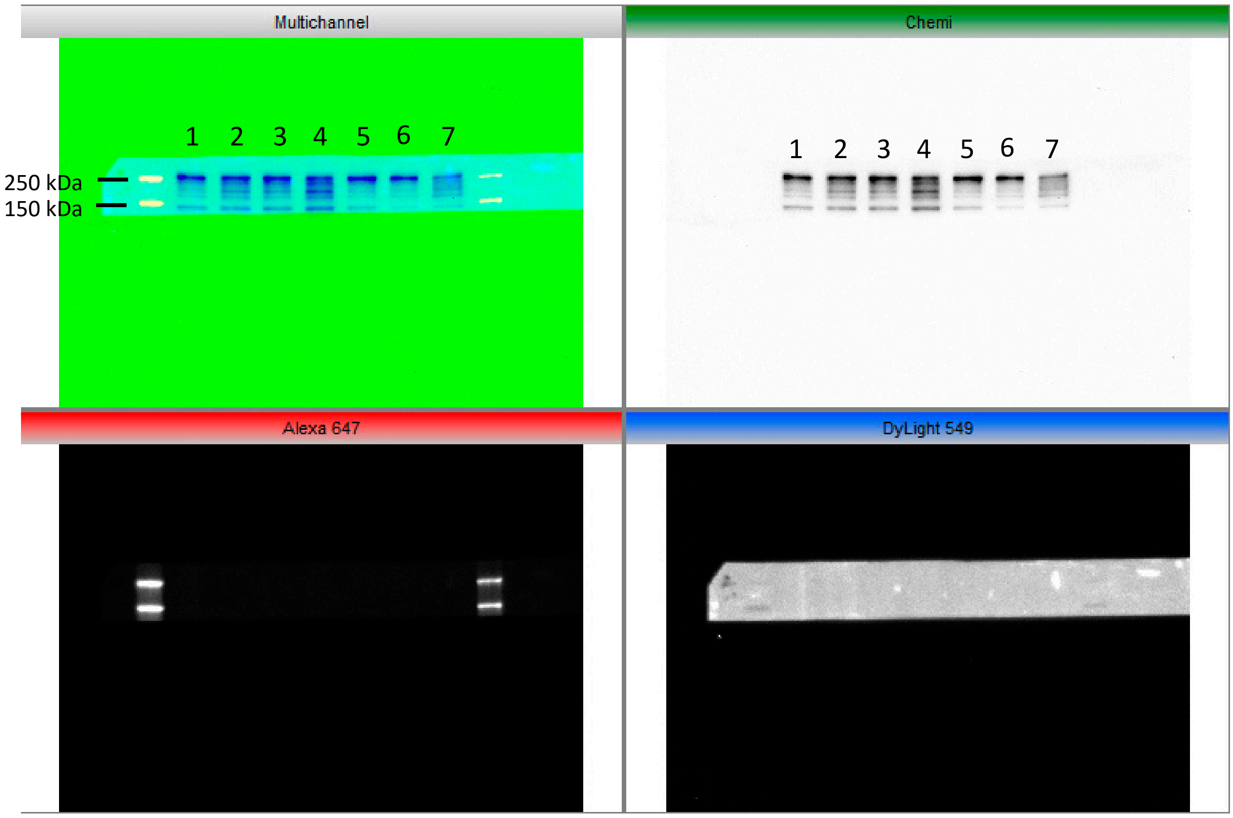

2. Akt (~ 60kDa)

1 – K562 + DMSO T0
2 – K562 + DMSO 4h
3 – K562 + DMSO 8h
4 - K562 + DMSO 16h
5 – K562 + BK124.1 4h
6 – K562 + BK124.1 8h
7 – K562 + BK124.1 16h

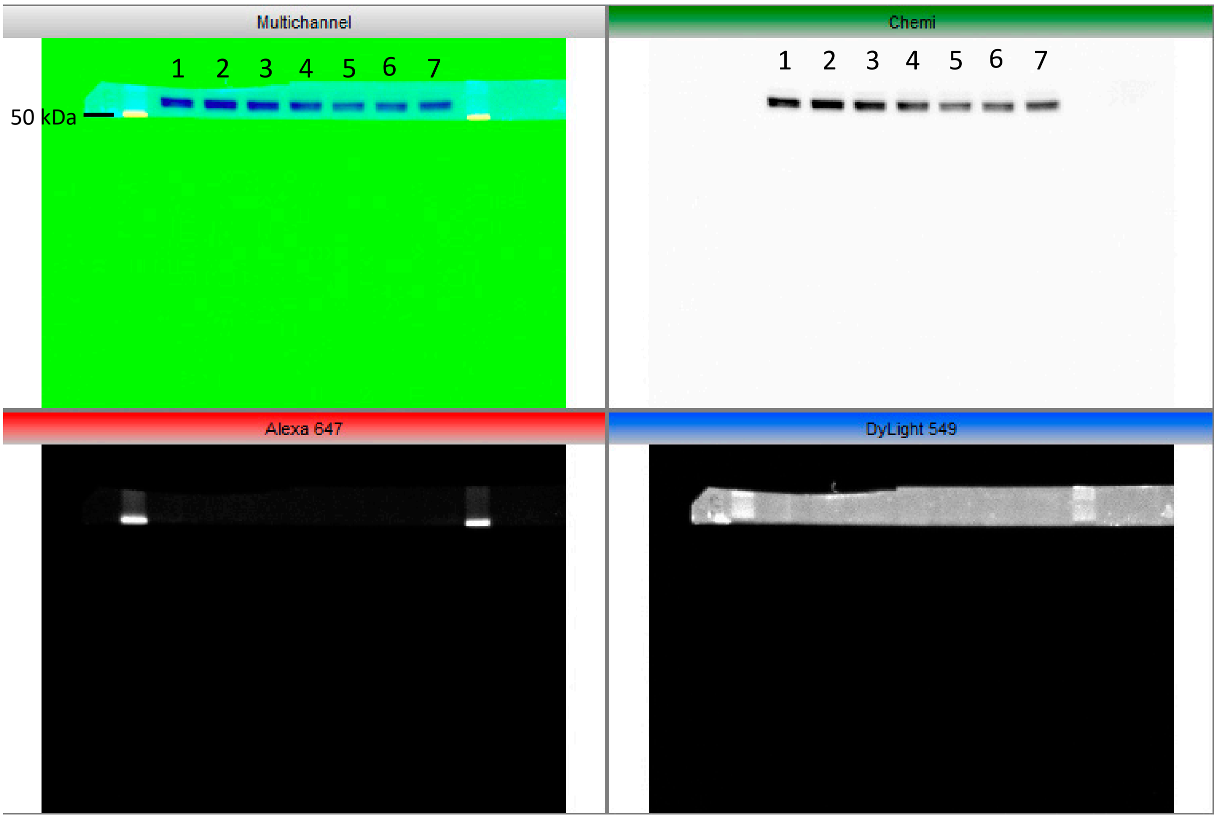

3. STAT5 (~ 60 kDa)

1 – K562 + DMSO T_0
2 – K562 + DMSO 4h
3 – K562 + DMSO 8h
4 - K562 + DMSO 16h
5 – K562 + BK124.1 4h
6 – K562 + BK124.1 8h
7 – K562 + BK124.1 16h

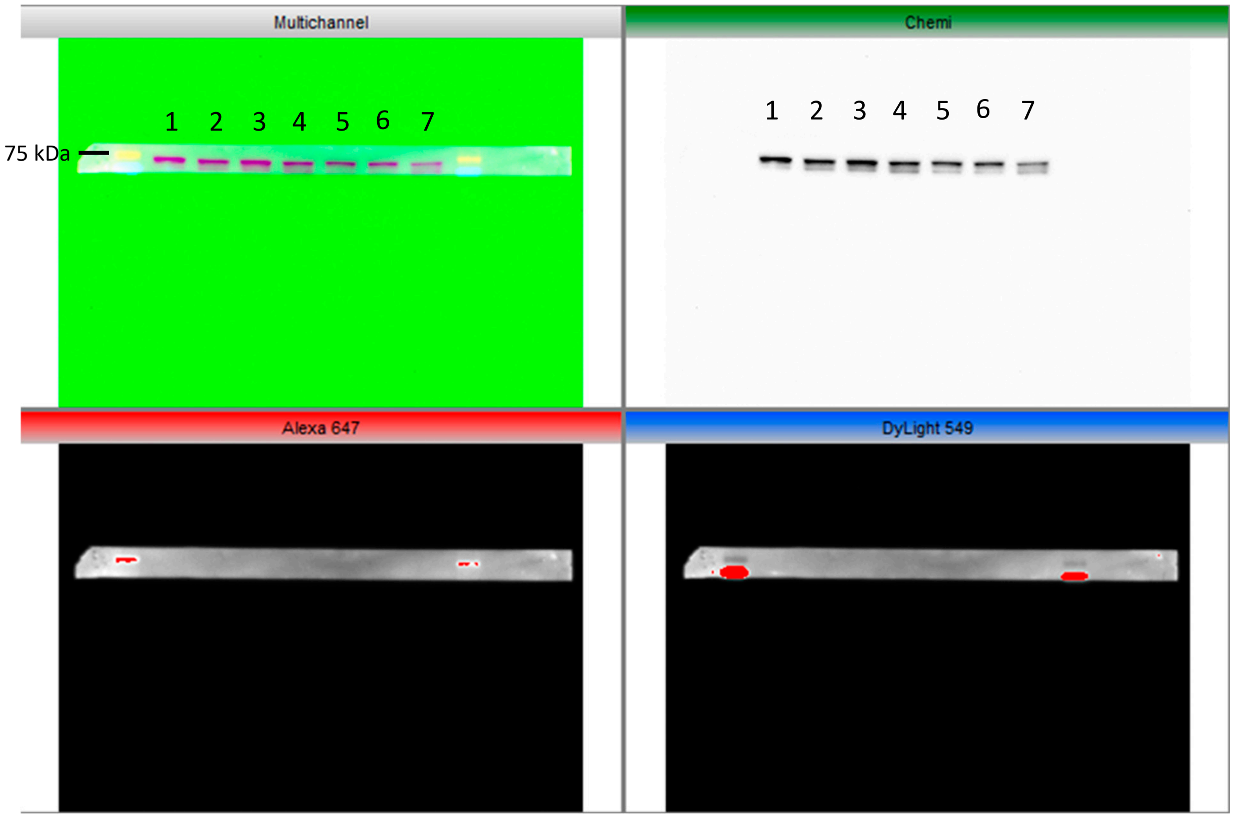

4. B-actin (~ 45 kDa)

1 – K562 + DMSO T_0
2 – K562 + DMSO 4h
3 – K562 + DMSO 8h
4 - K562 + DMSO 16h
5 – K562 + BK124.1 4h
6 – K562 + BK124.1 8h
7 – K562 + BK124.1 16h

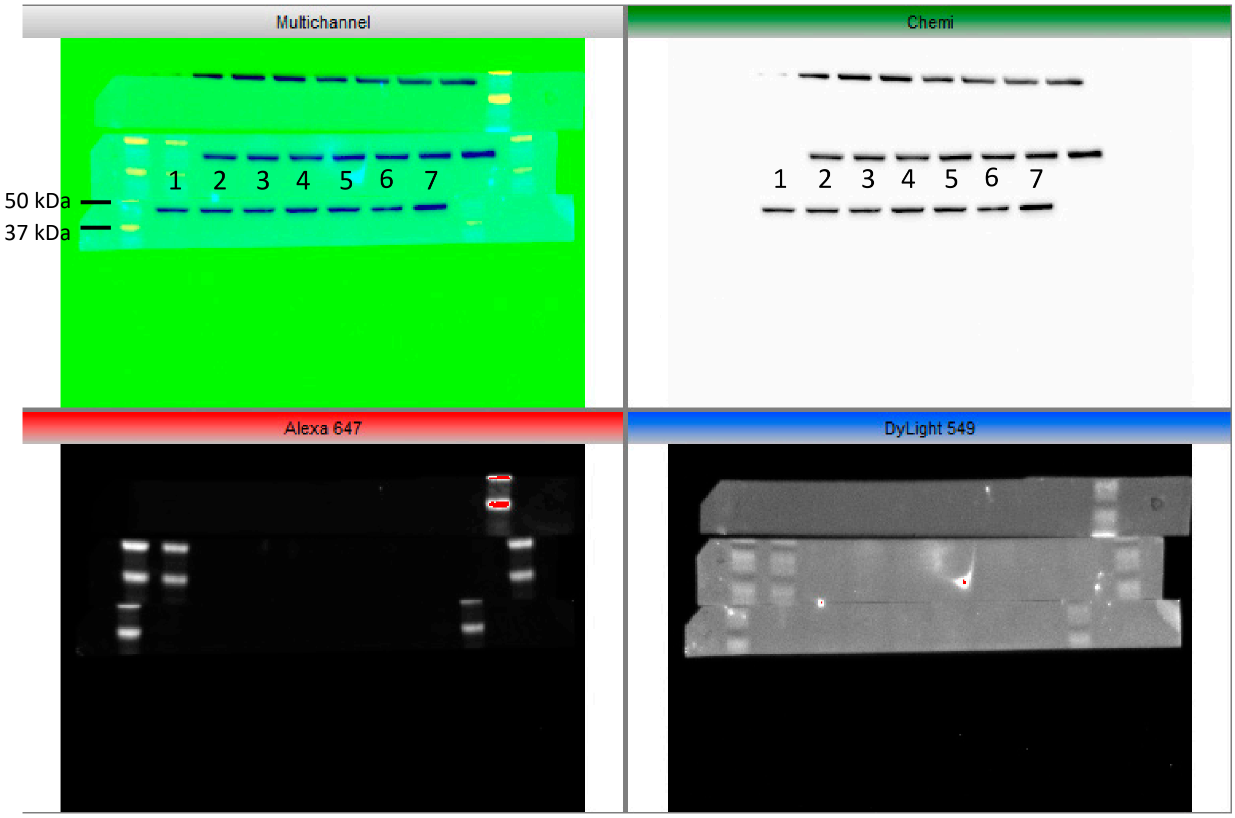

Figure S3. Whole blots for Western blots in Figure 2E (p65).

p65 (~ 65 kDa)

1 – K562 + BK124.1 4h

2 – K562 + BK124.1 8h

3 – K562 + BK124.1 16h

4 – K562 + DMSO T0

5 – K562 + DMSO 4h

6 – K562 + DMSO 8h

7 - K562 + DMSO 16h

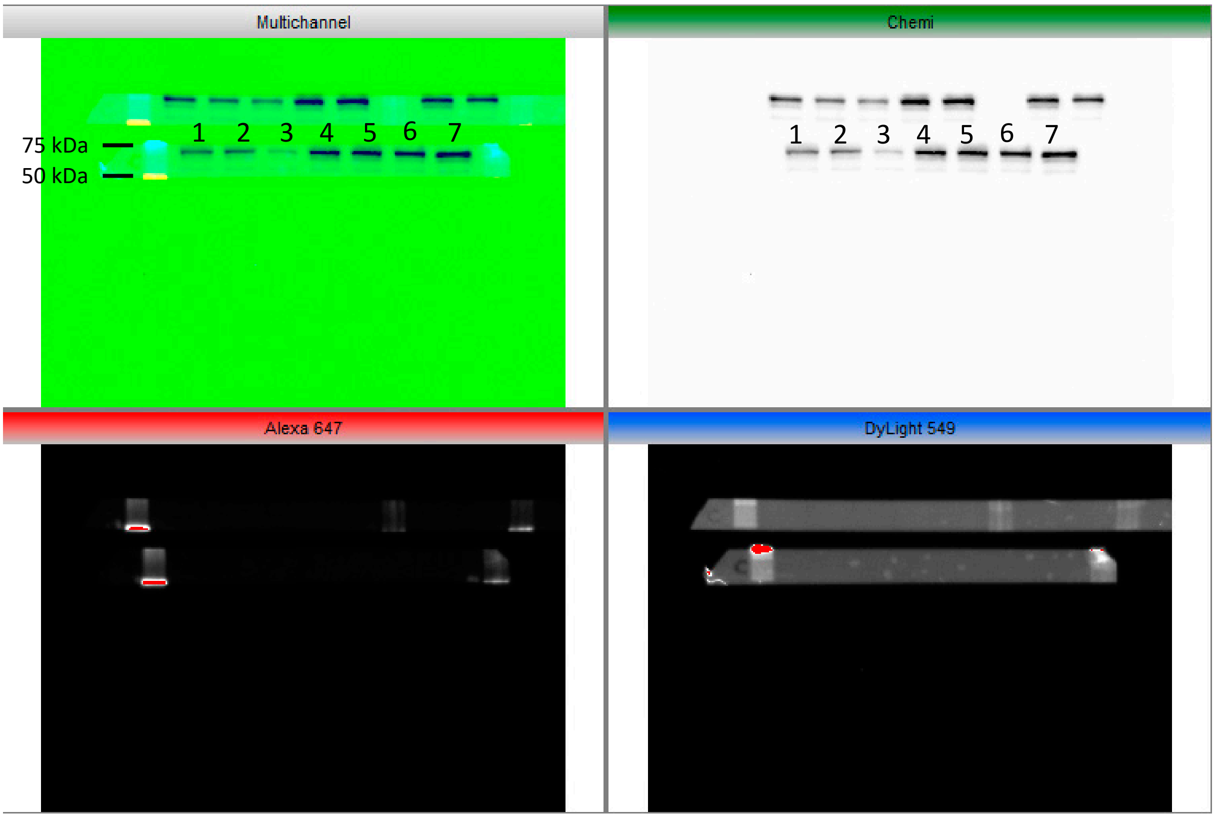

B-actin (~ 45 kDa)

1 – K562 + BK124.1 4h

2 – K562 + BK124.1 8h

3 – K562 + BK124.1 16h

4 – K562 + DMSO T0

5 – K562 + DMSO 4h

6 – K562 + DMSO 8h

7 - K562 + DMSO 16h

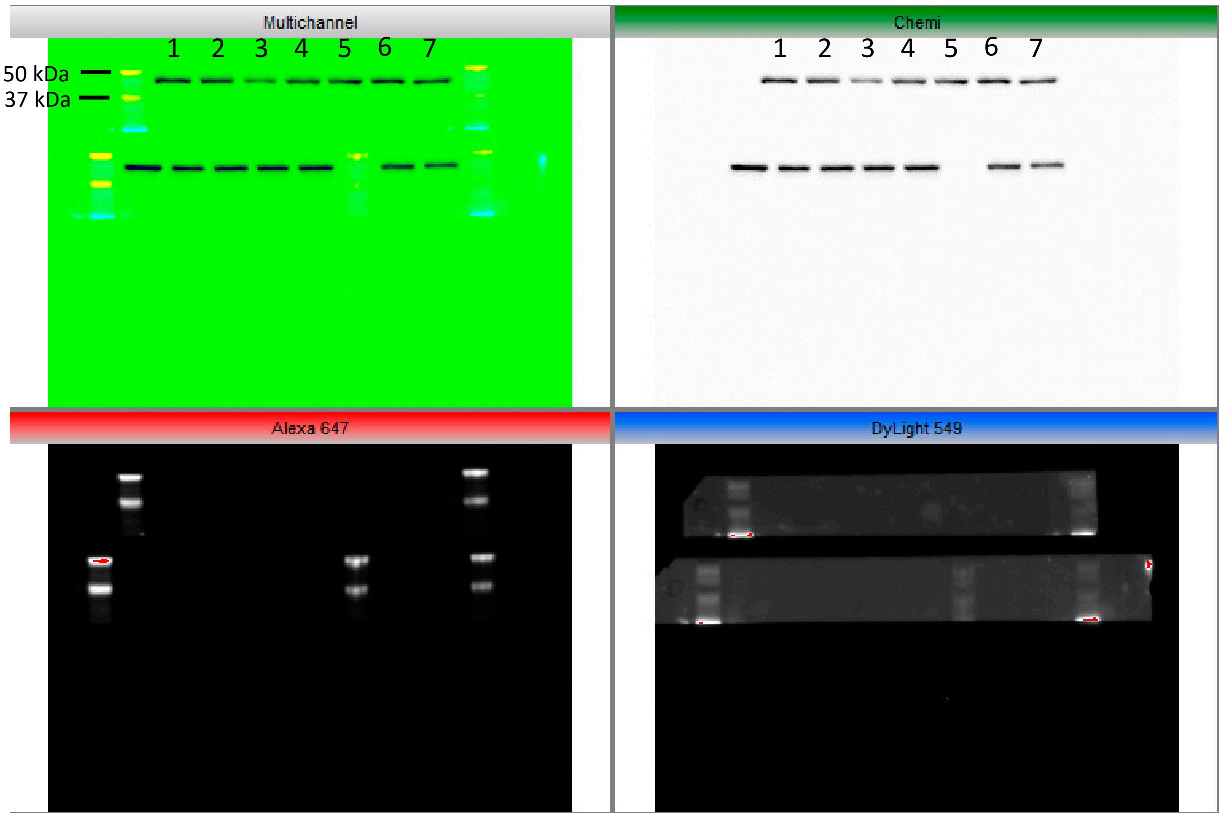

Figure S4. Whole blots for Western blots in Figure 2E mTOR (~ 289 kDa)

1 – K562 + BK124.1 4h

2 – K562 + BK124.1 8h

3 – K562 + BK124.1 16h

4 – K562 + DMSO T0

5 – K562 + DMSO 4h

6 – K562 + DMSO 8h

7 - K562 + DMSO 16h

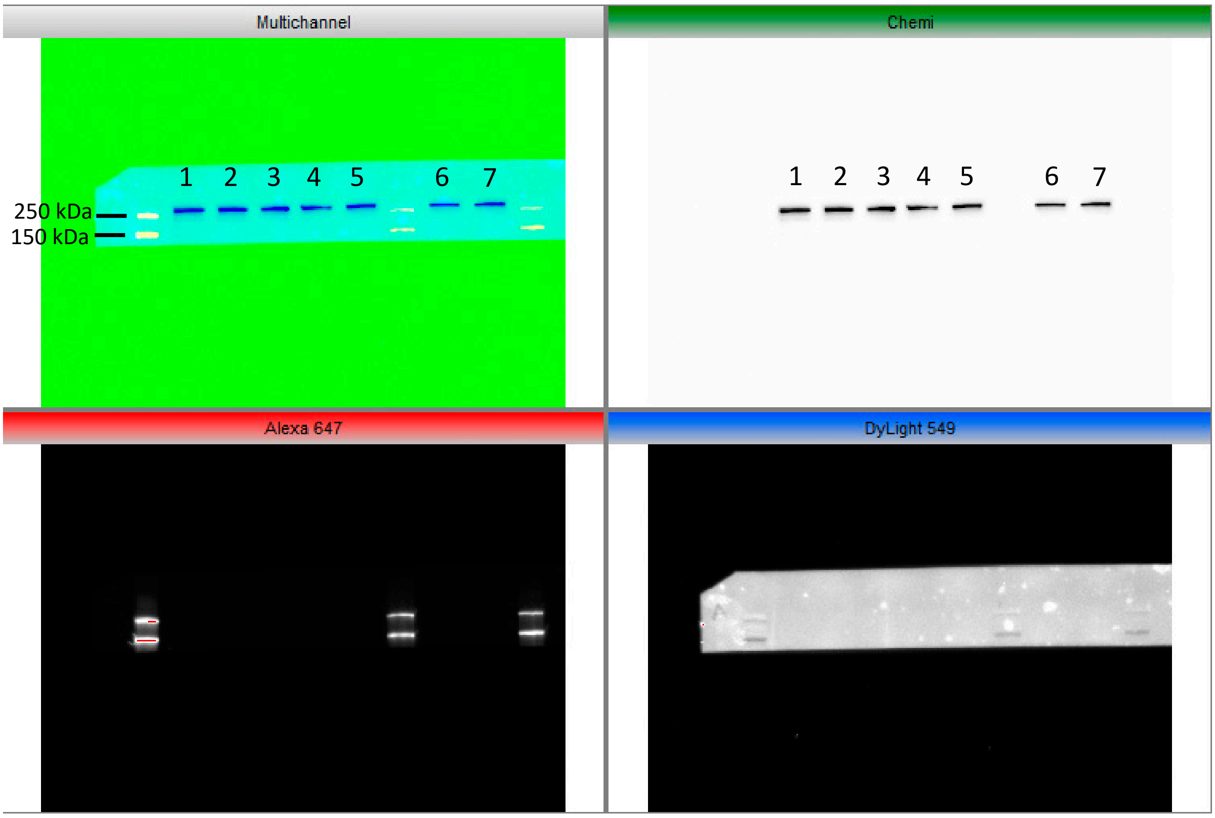

B-actin (~ 45 kDa)

1 – K562 + BK124.1 4h

2 – K562 + BK124.1 8h

3 – K562 + BK124.1 16h

4 – K562 + DMSO T0

5 – K562 + DMSO 4h

6 – K562 + DMSO 8h

7 - K562 + DMSO 16h

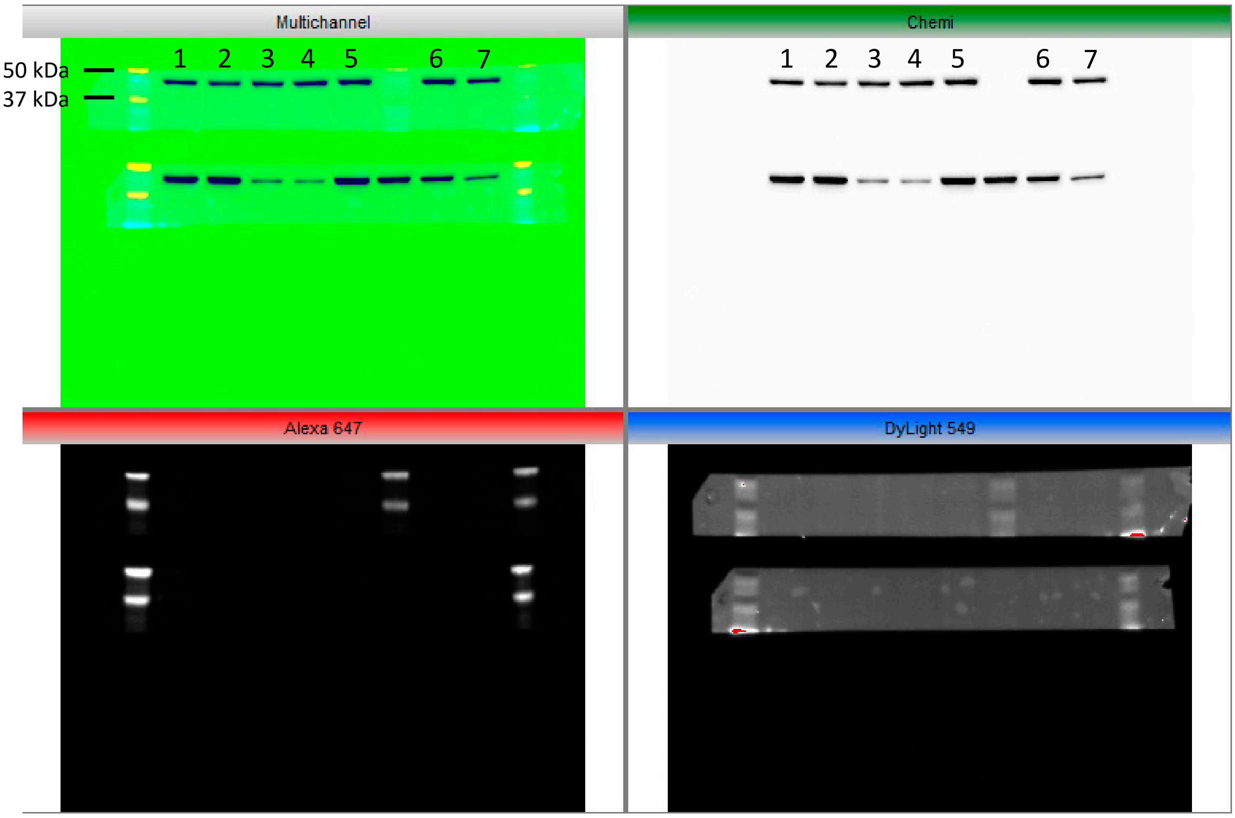

Figure S5. Whole blots for Western blots in Figure 2H.

P21 (~ 21 kDa)

1 – K562 + DMSO T_0
2 – K562 + DMSO 4h
3 – K562 + DMSO 8h
4 - K562 + DMSO 16h

5 – K562 + BK124.1 4h
6 – K562 + BK124.1 8h
7 – K562 + BK124.1 16h

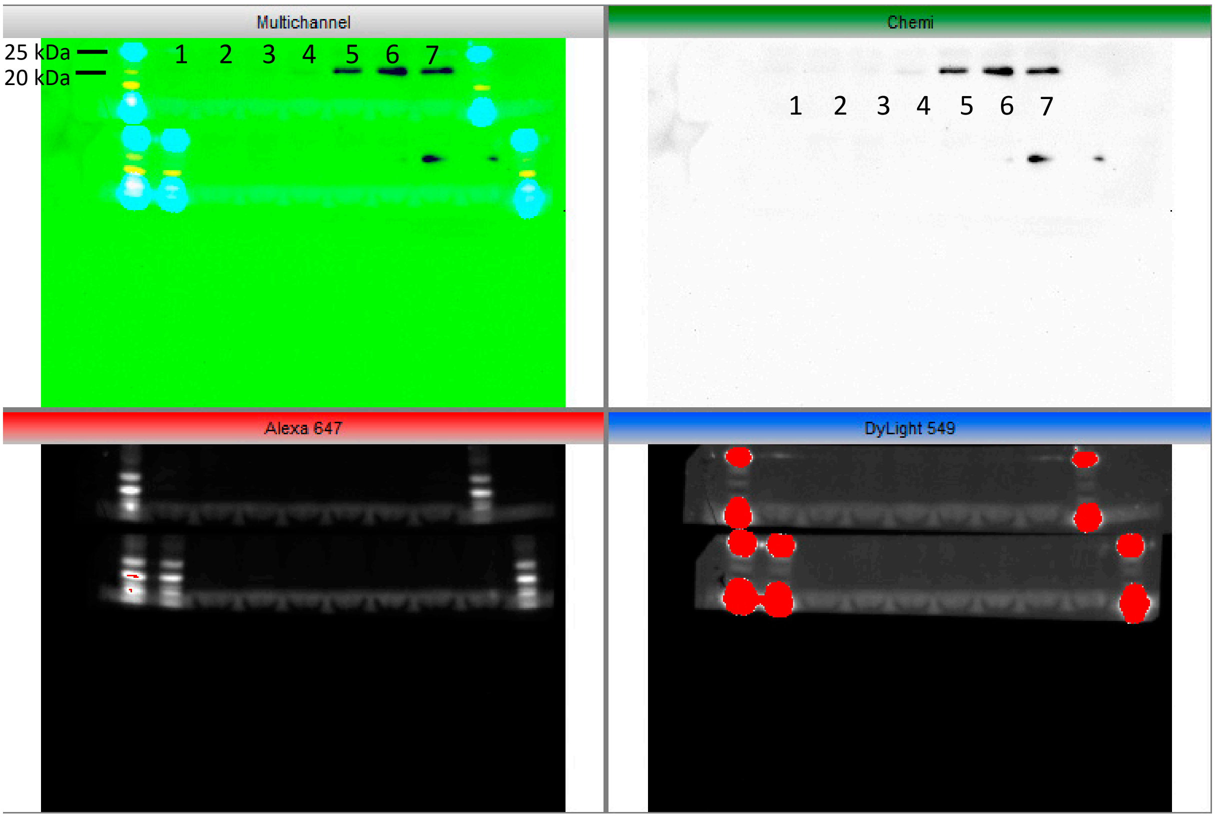

FOXO3a (~ 80 kDa)

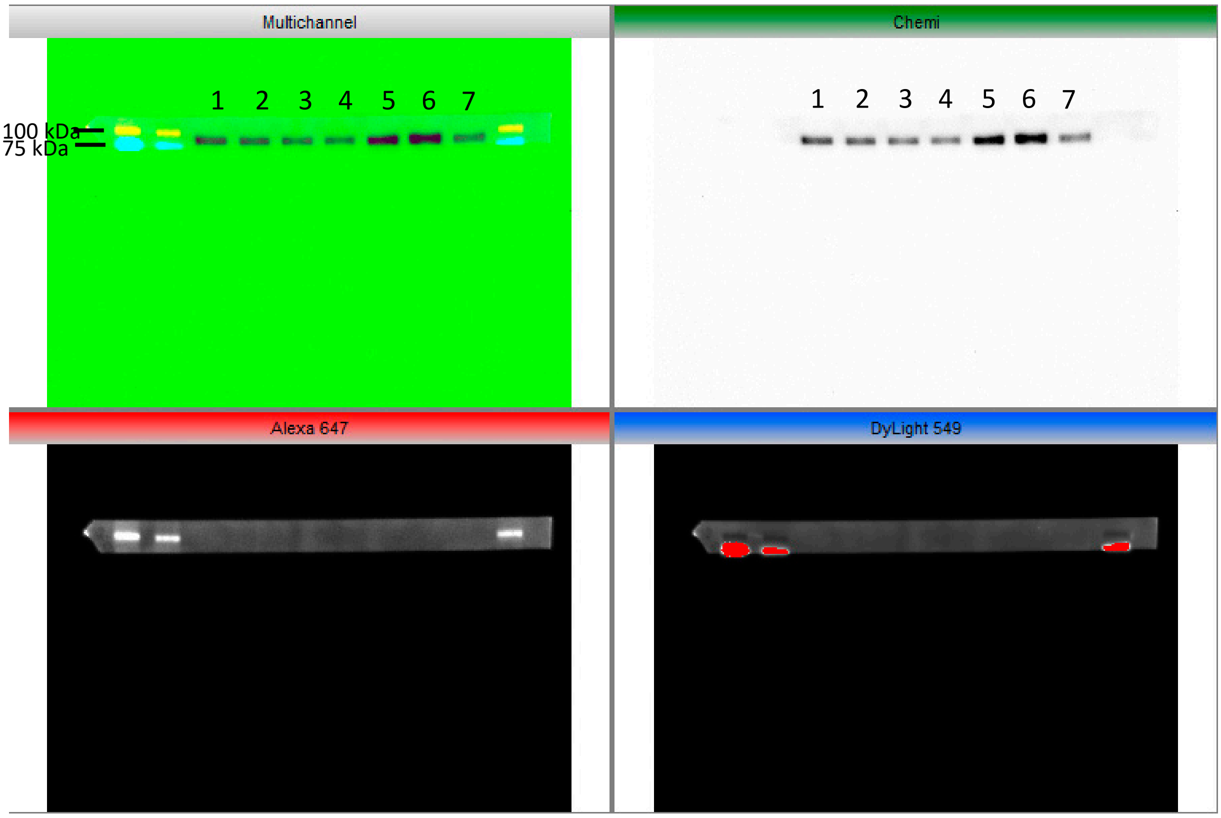

1 – K562 + DMSO T_0

2 – K562 + DMSO 4h

3 – K562 + DMSO 8h

4 - K562 + DMSO 16h

5 – K562 + BK124.1 4h

6 – K562 + BK124.1 8h

7 – K562 + BK124.1 16h

B-aktyna (~ 45 kDa)

1 – K562 + DMSO T_0
2 – K562 + DMSO 4h
3 – K562 + DMSO 8h
4 - K562 + DMSO 16h
5 – K562 + BK124.1 4h
6 – K562 + BK124.1 8h
7 – K562 + BK124.1 16h

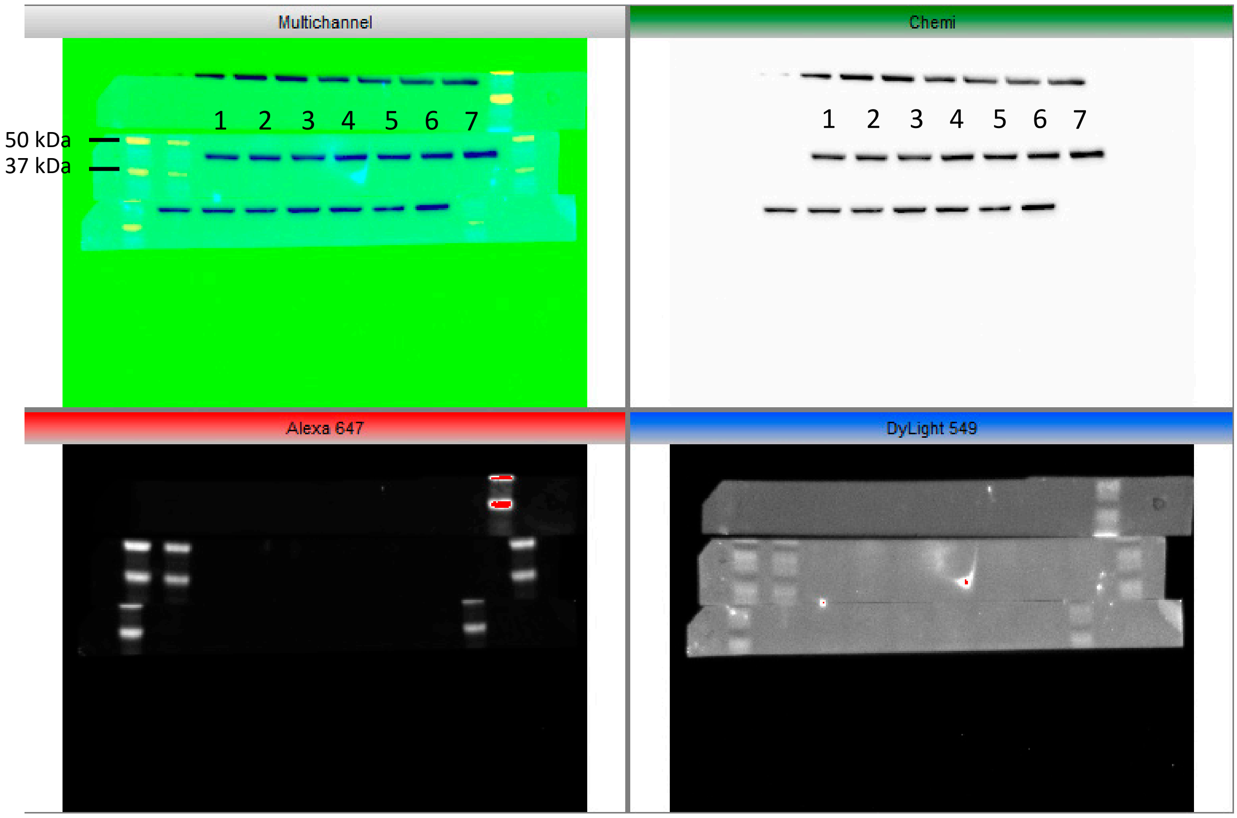
Multichannel
Chemi
1 2 3 4 5 6 7
50 kDa
37 kDa
1 2 3 4 5 6 7
Alexa 647
DyLight 549

Figure S6. Whole blots for Western blots in Figure 2K.

FOXO3a (~ 80 kDa)

1 – K562 + DMSO cytoplasm
2 – K562 + 5 uM BK124.1 cytoplasm
3 – K562 + DMSO nucleus
4 - K562 + 5 uM BK124.1 nucleus

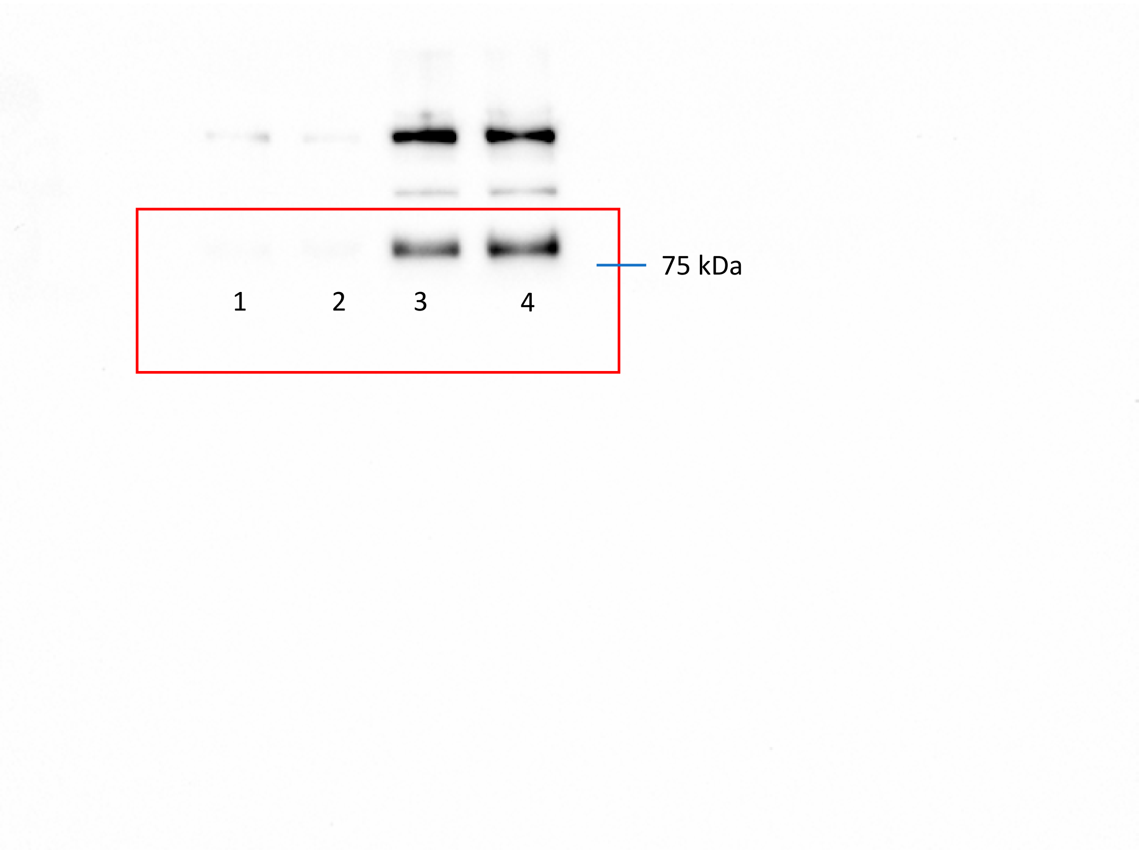

P21 and LMNB1 (~ 21 kDa and ~ 67 kDa respectively)

1 – K562 + DMSO cytoplasm
2 – K562 + 5 uM BK124.1 cytoplasm
3 – K562 + DMSO nucleus
4 - K562 + 5 uM BK124.1 nucleus

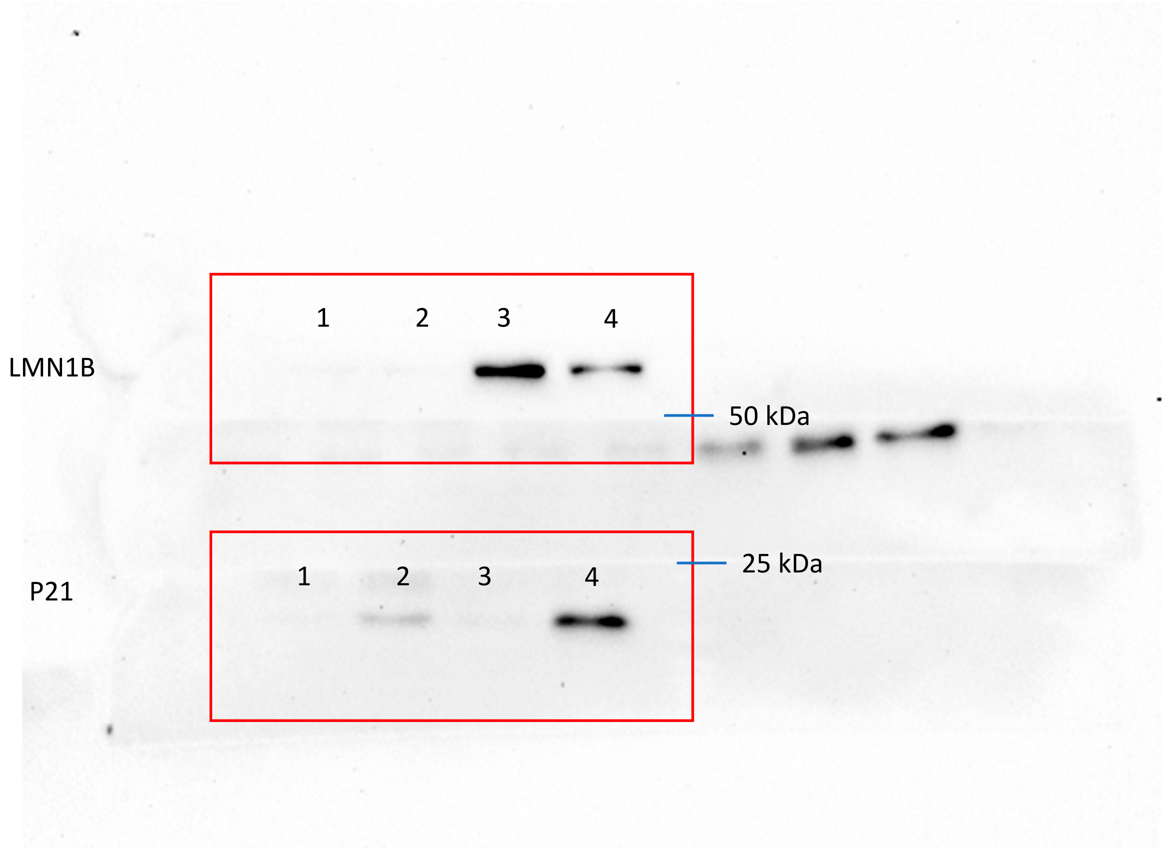

B-Actin (~ 45 kDa)

1 – K562 + DMSO cytoplasm
2 – K562 + 5 uM BK124.1 cytoplasm
3 – K562 + DMSO nucleus
4 - K562 + 5 uM BK124.1 nucleus

Additional file 2: Supplementary methods

Supplementary methods

Antibodies

The antibodies (Ab) used in the flow cytometry and immunoblotting experiments are listed according to the targeted protein and the vendor. Antibodies: BD Transduction Laboratories - p38 (ref. 612288), p-p38 (ref. 612289); Cell Signalling Technologies - c-Abl (ref. 2862), Akt (pan) (C67E7) (ref. 4691), STAT5 (ref. 9363), mTOR (7C10) (ref. 2983), NF-κB p65 (D14E12) XP® (ref. 8242), β-Actin (8H10D10) (ref. 3700), FOXO3a (75D8) (ref. 2497), p21 Waf1/Cip1 (12D1) (ref. 2947), p-ERK1/2 (ref. 9101), ERK1/2, (ref. 9102); Santa Cruz Biotechnology - Lamin B1 (A11) (ref. sc-377000), JNK, (ref. sc-7345), p-JNK (ref. sc-6254); Sigma - α-tubulin (ref. T-6074). Biolegend, San Diego, CA, USA - CD34 (CD34-APC), CD38 (anti-human CD38-PE).

BK124.1 preparation for injection

In the first step, 50 µL of Solutol HS 15 and 50 µL of anhydrous ethanol 99.8% were added to 1 mg of BK124.1, to obtain a solution of 50% Solutol HS 15/50% ethanol. This solution was shaken (1-2 h) at 30-40 °C until BK124.1 was completely dissolved. This solution was stable for 48 hours at temperatures between 30-36 °C. This concentrate is sticky, has poor filtration and portioning properties, and so cannot be administered intravenously or intraperitoneally without dilution. Therefore, in the second step, the prepared concentrate was diluted with *aqua pro injectione* to obtain a concentration of 10% Solutol HS 15/10% ethanol. The diluted solution was fluid, convenient for filtration and for portioning. This solution was sterilized by membrane filtration through a 0.2 µm filter (Minisart PES; Sartorius, Goettingen, Germany), pipetted into sterile 2 mL tubes, and then stored at room temperature for 48 hours until used for injection in animals.

Isolation and culture of CD34$^+$ cells from CML patients

The blood samples obtained from CML patients were diluted 1:1 with a balanced salt solution containing: 0.01% anhydrous D-glucose (Sigma-Aldrich, St. Louis, MO, USA), 5 µM $CaCl_2 \cdot 2H_2O$ (VWR,

Radnor, PA, USA), 98 µM $MgCl_2 \cdot 6 H_2O$ (VWR, Radnor, PA, USA), 0.54 mM KCl (VWR, Radnor, PA, USA), 14.5 mM Tris (Sigma-Aldrich, St. Louis, MO, USA), pH 7.6 in 0.9% saline (VWR, Radnor, PA, USA) and centrifuged on Ficoll-Paque Plus (GE Healthcare, Chicago, IL, USA). Peripheral blood mononuclear cells (PBMCs) from the plasma/Ficoll interface were washed twice with the above-mentioned balanced salt solution. Next, we isolated $CD34^+$ cells (EasySep™ Human CD34 Positive Selection Kit II, StemCell Technologies, Vancouver, Canada), counted them and assessed viability with Muse Cell Analyzer (Merck Millipore, Burlington, MA, USA). $CD34^+$ were cultured in StemPro™-34 SFM medium (Thermo Fisher Scientific, Waltham, MA, USA) with fresh addition of recommended cytokines: IL-3 (50 ng/mL), GM-CSF (25 ng/mL) and SCF (100 ng/mL) (PeproTech, London, UK). For BK124.1 experiments, cells were always pre-cultured for 24 hours in the cell culture incubator at 37 °C and 5% CO_2. The purity of the live $CD34^+$ fraction was assessed by flow cytometry with anti-human CD34-APC antibody, compared with an appropriate isotype control (Biolegend, San Diego, CA, USA), and propidium iodide (Sigma-Aldrich, St. Louis, MO, USA).

Measurement of purity and apoptosis in $CD34^+/CD38^-$ cells

$CD34^+$ cells isolated from peripheral blood of CML patients were seeded at a concentration of 2.5×10^5 cells/mL in a 24-well plate and cultured for 24 hours in complete medium. For purity analysis cells were harvested after 24 hours and stained with anti-human CD34-APC antibody, anti-human CD38-PE (Biolegend, San Diego, CA, USA) and propidium iodide according to the manufacturers' protocols. For apoptosis analysis, cells were treated with 2.5 µM or 5 µM BK124.1, or DMSO, harvested after 24 hours, and stained with anti-human CD34-APC antibody, anti-human CD38-PE and Annexin V-FITC kit (BD, Franklin Lakes, NJ, USA) according to the manufacturer's' protocol. Appropriate isotype controls were included. In both cases stained cells were immediately analyzed on a BD LSRFortessa flow cytometer and analyzed with FlowJo software (BD, Franklin Lakes, NJ, USA). Purity was analyzed with an exclusion of doublets on live cells only. Analysis of apoptosis was performed with doublet exclusion and for each population of cells ($CD34^+/CD38^+$ or $CD34^+/CD38^-$) separately.

Cell viability MTT test

Cells were seeded in 96-well plates at 7×10^3 cells/well in 50 µL of complete medium (for K562 or K562-MDR1) or at 50×10^3 cells/well in 50 µL of complete medium ($CD34^+$ cells). After 24 hours, 50 µL of medium with the appropriate drug concentration was added to each well. The final DMSO concentration per well was 1% for K562 and K562-MDR1 and 0.1% for $CD34^+$ cells. After 48 hours, 20 µL of Tetrazolium Bromide (Sigma-Aldrich, St. Louis, MO, USA) at a concentration of 5 mg/mL was added to each well. Cells were incubated for 2-3 hours in a cell culture incubator, and then 10% SDS with 1% HCl was added to each well to lyse the cells and dissolve the formazan crystals. Plates were

incubated overnight at 37 °C and analyzed using an iMark Microplate absorbance reader (Bio-Rad, Hercules, CA, USA). Results were calculated relative to control cells treated with DMSO only.

Measurement of apoptosis

K562 or K562-MDR1 cells were seeded in a 6-well plate at a concentration of 1-2 × 10^5 cells/mL. $CD34^+$ cells were plated at a concentration of 2.5 × 10^5 cells/mL in a 24-well plate. After 24 hours of pre-incubation, the test compound dissolved in DMSO (Sigma-Aldrich, St. Louis, MO, USA) was added. After a further 24 hours, cells were harvested and stained according to the manufacturer's protocol using the Annexin V-FITC kit for the detection of apoptosis (BD, Franklin Lakes, NJ, USA). Stained cells were immediately analyzed on a BD FACSCalibur using CellQuest software (BD, Franklin Lakes, NJ, USA).

Cell cycle analysis

Cells were seeded at a concentration of 1-2 × 10^5 cells/mL in a 6-well plate. After 24 hours of pre-incubation, BK124.1 in DMSO was added to the cells (v_{DMSO}/v_{medium}), and incubated for 24 or 48 hours. After incubation, cells were washed twice with ice-cold PBS (Mg^{2+} and Ca^{2+}-free), resuspended in ice-cold 70% ethanol, and placed at -20 °C for 24 hours. After this time, the ethanol was washed off, the cells were resuspended in 50 µg/mL propidium iodide (Sigma-Aldrich, St. Louis, MO, USA) with 50 µg/L DNAse free RNase in PBS and incubated at 37 °C for 30 minutes. The cell cycle was analyzed by flow cytometry on a FACSCalibur cytometer (Becton Dickinson, Franklin Lakes, NJ, USA) using ModFit LT 3.2 software (Verity Software House, Topsham, ME, USA).

Flow cytometry analysis of CD243 (P-glycoprotein)

Cells were seeded at a concentration of 1-2 × 10^5 cells/mL in a 6- or 24-well plate. After 24 hours of pre-incubation, DMSO or test compounds in DMSO were added. The cells were then incubated for 24 hours. After this time, cells were harvested, washed with 0.5% BSA in PBS (Mg^{2+} and Ca^{2+} free), and labeled for 30 minutes on ice with FITC Mouse Anti-Human P-glycoprotein (CD243) clone 17F9 (BD, Franklin Lakes, NJ, USA) or an appropriate isotype control. Following incubation with the antibody, cells were stained with propidium iodide, washed with PBS and analyzed on a BD FACSCalibur flow cytometer using CellQuest software (BD, Franklin Lakes, NJ, USA).

K562 in BK124.1 long term experiment (6 months)

K562 cells were cultured in 18-well plate with or without 0,1% DMSO (v/v) and with 1 µM or 2 µM BK124.1 in 0,1% DMSO (v/v). Medium was renewed every 2-3 days. Cells were passaged when they reach concentration of 10^6 cells/ml. After 6 months cells were collected for MTT cytotoxicity assessment, RNA and protein analysis. RNA was isolated with High Pure RNA Isolation Kit (Roche, Basel,

Switzerland), reverse transcription was performed with High-Capacity RNA-to-cDNA™ Kit (Applied Biosystems, Waltham, MA, USA) in Mastercycler® Nexus (Eppendorf, Hamburg, Germany). Applied Biosystems™ TaqMan™ Expression Assay probes used for real-time PCR: GAPDH (Hs02786624_g1), ABCB1 (Hs00184500_m1). RT qPCR was performed using Applied Biosystems 7900HT Fast Real-Time PCR System. P-glycoprotein analysis was done using flow cytometry as described in materials and methods; MTT cytotoxicity assessment was prepared as described in materials and methods; MTT results were plotted and IC_{50} was calculated using Dr Fit software version 1.042 64-bit (The MIT license 2015).
